# Promising Drug Repurposing Candidates Targeting Free-Living Amoebae: A Systematic and Critical Review of Laboratory-Based Evidence

**DOI:** 10.3390/pathogens15030294

**Published:** 2026-03-07

**Authors:** Beni Jequicene Mussengue Chaúque, Luiza Bernardes Chagas, Thaisla Cristiane Borella da Silva, Denise Leal dos Santos, Luciano Palmeiro Rodrigues, Lucile da Silva Lins Baía, Manoella Kessler Gomes Rodrigues, Guilherme Brittes Benitez, Thais Lemos Mendes, Hellen Kempfer Philippsen, Luciana Dalla Rosa, Fabrício Souza Campos, Marilise Brittes Rott, Régis Adriel Zanette, José Roberto Goldim

**Affiliations:** 1 Master’s Program in Clinical Research, Hospital de Clínicas de Porto Alegre, Porto Alegre 90035-903, RS, Brazil; delealsantos1970@gmail.com (D.L.d.S.); lucianorodrigues@hcpa.edu.br (L.P.R.); jgoldim@hcpa.edu.br (J.R.G.); 2 Postgraduate Program in Biological Sciences—Pharmacology and Therapeutics, Federal University of Rio Grande do Sul, Porto Alegre 90010-150, RS, Brazil; integrittapet@gmail.com (L.d.S.L.B.); thaismendes96@hotmail.com (T.L.M.); regnitro@yahoo.com.br (R.A.Z.); 3 Center of Studies in Science and Technology (NECET), Biology Course, Universidade Rovuma, Lichinga P.O. Box 04, Niassa, Mozambique; 4 Faculty of Pharmacy, Federal University of Rio Grande do Sul, Porto Alegre 90010-150, RS, Brazil; luizabch@hotmail.com; 5 Protozoology Laboratory, Microbiology Immunology and Parasitology Department, Basic Health Sciences Institute, Federal University of Rio Grande do Sul, Porto Alegre 90010-150, RS, Brazil; thaislacristiane@gmail.com (T.C.B.d.S.); marilise.rott@ufrgs.br (M.B.R.); 6 Physiotherapy Course, Federal University of Rio Grande do Sul, Porto Alegre 90010-150, RS, Brazil; 7 School of Medicine, Pontifícia Universidade Católica do Rio Grande do Sul (PUCRS), Porto Alegre 90610-970, RS, Brazil; manoellakessler18@gmail.com; 8 Industrial and Systems Engineering Graduate Program, Polytechnic School, Pontifical Catholic University of Parana (PUCPR), Curitiba 80215-901, PR, Brazil; guilherme.benitez@pucpr.br; 9 Socio-Environmental and Water Resources Institute, Federal Rural University of the Amazon (UFRA), Belém 66077-830, PA, Brazil; hellen.kempfer@ufra.edu.br; 10 Central Laboratory for Avian Disease Diagnosis, Department of Preventive Veterinary Medicine, Center for Rural Sciences, Universidade Federal de Santa Maria, Santa Maria 97105-900, RS, Brazil ; lucianadallarosa@gmail.com; 11 Laboratório de Bioinformática & Biotecnologia, Instituto de Ciências Básicas da Saúde, Federal University of Rio Grande do Sul, Porto Alegre 90010-150, RS, Brazil; camposvet@gmail.com; 12 Department of Public & Ecosystem Health, College of Veterinary Medicine, Cornell University, Ithaca, NY 14853, USA

**Keywords:** drug repurposing, *Acanthamoeba* keratitis, granulomatous amoebic encephalitis, primary amoebic meningoencephalitis, in vitro, in vivo

## Abstract

Devastating or nearly invariably fatal infections caused by free-living amoebae (FLA), including *Acanthamoeba* keratitis (AK), granulomatous amoebic encephalitis (GAE), and primary amoebic meningoencephalitis (PAM), remain a significant public health concern, driven by increasing case numbers, geographic expansion, and the lack of approved, effective, and safe treatments. Despite decades of research, no new drugs have been successfully approved, highlighting the severe limitations of de novo drug development for these infections, particularly for GAE and PAM, largely due to the challenges of conducting clinical trials for these rare and rapidly lethal diseases. In this context, drug repurposing represents a cost-effective and promising strategy to accelerate therapeutic advances and overcome key bottlenecks of conventional drug development. Accordingly, we conducted a systematic review of in vitro studies and animal models of AK, GAE, and PAM reported in indexed databases to identify promising drug repurposing candidates against FLA infections. After screening 23,624 records, 112 studies were included in the analysis. Overall, 2726 drugs and drug combinations, spanning 865 pharmacological classes and approved for 565 therapeutic indications, were assessed for their repurposing potential. Among these, 166 compounds showed substantial trophocidal activity (≥IC_50_) at potentially translatable concentrations (≤10 µM), including six with additional cysticidal activity. In vitro, four compounds were active against *Balamuthia mandrillaris*, 44 against *Acanthamoeba* spp. (three cysticidal), and 115 against *Naegleria* spp. (three cysticidal). In in vivo studies, sulfadiazine and rifampicin were effective as preventive or early monotherapies for GAE. For AK, the combination of polyhexamethylene biguanide, neomycin, and atropine, as well as voriconazole and nitazoxanide monotherapies, showed the greatest promise. In PAM, azithromycin alone or in combination with amphotericin B emerged as the most promising therapeutic options. Further studies are required to advance the clinical translatability of these findings. To the best of our knowledge, this work provides the first comprehensive and integrated synthesis of repurposable drug candidates against FLA infections.

## 1. Introduction

Free-living amoebae (FLA) are a diverse group of amphizoic protists comprising the genera *Naegleria*, *Acanthamoeba*, *Balamuthia*, *Vermamoeba*, and *Sappinia*. These microorganisms are ubiquitous in both natural and engineered environments, including solid matrices (e.g., soil, dust, sediment, sludge) [1], various water sources (e.g., chlorinated, bottled, and permafrost water), sewage, and air [2,3,4]. In their trophozoite form (whether flagellated or not) they feed, reproduce, and can exert pathogenic effects. Under adverse environmental conditions, they initiate stress response pathways that lead to the formation of cysts, resilient structures characterized by a double wall containing cellulose [5,6]. These cysts confer significant resistance to FLA against a range of physicochemical stressors, such as radiation, heat, dehydration, freezing, chlorine, and salinity, as well as antimicrobial agents [7,8].

These organisms, particularly *Naegleria fowleri*, *Acanthamoeba* spp., *Balamuthia mandrillaris*, *Vermamoeba vermiformis*, and *Sappinia pedata*, are opportunistic pathogens capable of causing severe and often fatal infections in humans and non-human animals, including primary amoebic meningoencephalitis (PAM), granulomatous amoebic encephalitis (GAE), and *Acanthamoeba* keratitis [9,10]. Disseminated infections involving other organs (such as the liver, bones, skin, lungs, and sinuses) have also been reported, primarily affecting immunocompromised individuals and transplant recipients [11,12,13,14].

Although rare, infections such as PAM (caused by *N. fowleri* or, more rarely, *S. pedata*) and GAE (caused by *B. mandrillaris* or *Acanthamoeba* spp.) exhibit mortality rates exceeding 90%, mainly due to delayed diagnosis, the absence of effective and safe therapies, and the remarkable resilience of amoebic cysts [15,16,17].

Although substantial scientific efforts have been made across multiple fronts to identify promising compounds for the development of anti-FLA drugs, there remains a critical shortage of therapeutics that are both effective and safe for treating FLA infections [18,19,20,21,22,23,24,25]. Current treatment regimens are largely empirical, relying on combinations of non-specific chemical antimicrobials with inconsistent efficacy and significant toxicity [15,16,26].

To overcome these challenges, drug repurposing, defined as the therapeutic reapplication of existing drugs, has emerged as a promising strategy for identifying novel antiamoebic agents. This approach leverages existing pharmacological and toxicological data from already approved compounds, allowing for accelerated development pipelines and cost reduction [24,25].

In this context, with the aim of bridging the current knowledge gap, this systematic review compiles, analyzes, and critically synthesizes laboratory-based studies that evaluate the repositioning potential of various drugs against FLA. By focusing on both in vitro and in vivo models, the review aims to highlight the most promising candidates based on their anti-FLA activity and potency at low concentrations, with the ultimate goal of informing future translational research and guiding the development of more effective therapeutic protocols for these neglected yet deadly infections.

## 2. Materials and Methods

### 2.1. Review Question and Objectives

This review sought to answer the following questions: Which drugs, approved for the treatment of other diseases, have been evaluated for their amoebicidal activity in vitro or in vivo? Among these, which exhibit high trophocidal and/or cysticidal potency under ideal to moderate ADMET (Absorption, Distribution, Metabolism, Excretion, and Toxicity; IC_50_ ≤ 20 µM) conditions, and therefore possess strong potential for repurposing in the treatment of infections caused by free-living amoebae (FLA)? Accordingly, the objective of this study was to systematically identify approved drugs with high repurposing potential for the treatment of infections caused by FLA and to highlight candidates that warrant prioritization in future translational research.

### 2.2. Article Collection and Screening Procedure

The methodological procedures applied throughout all stages of article and data screening followed the PRISMA 2020 (Preferred Reporting Items for Systematic Reviews and Meta-Analyses) guidelines [27]. The review protocol was registered in the Protocols.io under DOI: 10.17504/protocols.io.ewov1kxz2gr2/v1, and the article’s PRISMA 2020 checklist is provided in File S1.

To maximize retrieval of relevant studies, the following broad search strategy was employed: “*Acanthamoeba* OR *Naegleria* OR *Balamuthia* OR *Vermamoeba*.” Searches were conducted in four databases (Web of Science, EMBASE, PubMed, and ProQuest). The consistency of search results across these platforms was independently verified by two authors. Formal searches were completed on June 11, 2024, with no restrictions on publication date.

**Figure 1:**
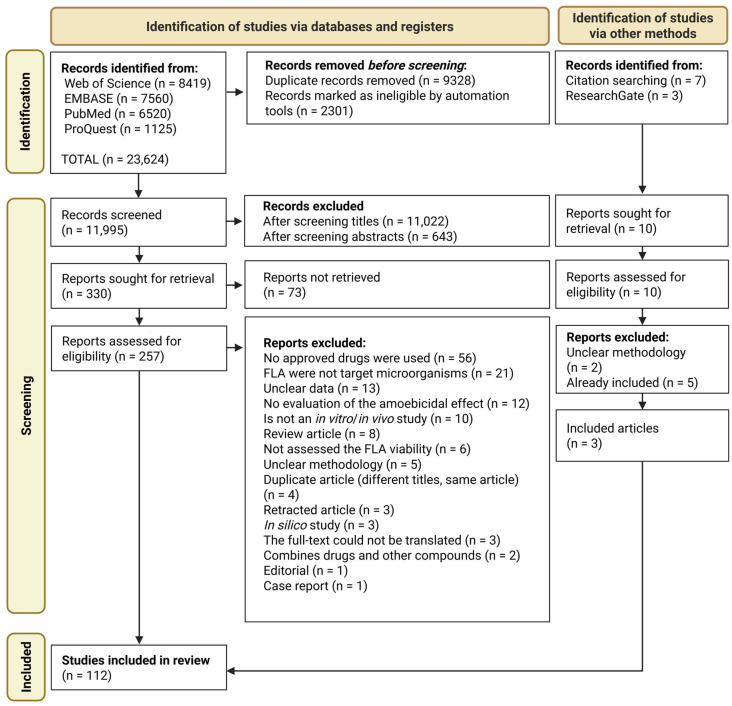
PRISMA flowchart depicting the retrieval and screening of studies on drug repositioning for free-living amoebae infections.

All retrieved records were subsequently subjected to the screening process (Figure 1) based on the inclusion and exclusion criteria detailed below.

• Inclusion and exclusion criteria: Articles were included if (1) the full text was available online; (2) they were written in English, Portuguese, Spanish, or another language that could be reliably translated using online tools; and (3) they reported findings from in vitro and/or in vivo evaluations of the amoebicidal activity of approved drugs against *Acanthamoeba*, *Naegleria*, *Balamuthia*, *Vermamoeba*, or *Sappinia*.
• Exclusion criteria: Articles were excluded if they (1) were reviews; (2) were not peer-reviewed (e.g., preprints); or (3) reported on compounds not approved as drugs.

Article screening was conducted independently and in parallel by at least two authors at each stage. Any disagreements were resolved through consensus meetings involving the dissenting authors and an additional reviewer.

After screening, the bibliographic references of the included articles were reviewed to identify any relevant literature not retrieved through direct database searches (Figure 1).

### 2.3. Data Extraction

The selected articles underwent data extraction to collect the following information: study reference details, nationality of all study authors, tested drug name, drug class, approved therapeutic indication, FLA species identification, FLA life stage (trophozoite or cyst), FLA density, effective drug concentration, exposure time (hours), mortality rate, half inhibitory concentration (IC_50_), half cytotoxic concentration in non-target mammalian cells (CC_50_), toxicity rate in non-target mammalian cells, and the mammalian cell line used for cytotoxicity assays.

Data extraction was initially performed by one author, followed by verification by a second and then a third author to ensure accuracy. Drug concentration values were converted to micromolar (µM) units when necessary.

### 2.4. Data Analysis

All statistical analyses were conducted using the R software 4.5.2 environment (R Foundation for Statistical Computing, Vienna, Austria). A customized script was developed to compute descriptive statistics for each compound, including mean, standard deviation, minimum, and maximum values for density, effective concentration, exposure time, and mortality-related parameters (including IC_50_). Data were grouped by drug name to ensure independent statistical summaries for each compound.

Standard deviations were calculated only when more than one valid observation was available; when a single observation was present, the standard deviation was set to zero to avoid overestimation of variability. This procedure ensured statistical robustness and consistency across compounds with unequal numbers of observations.

Following statistical processing, the dataset was cleaned, curated, and organized. A comprehensive analytical report was generated to document all data handling and statistical procedures, thereby ensuring transparency, reproducibility, and methodological reliability. All figures were generated using GraphPad Prism version 5 (GraphPad Software, San Diego, CA, USA).

## 3. Results

**Figure 2:**
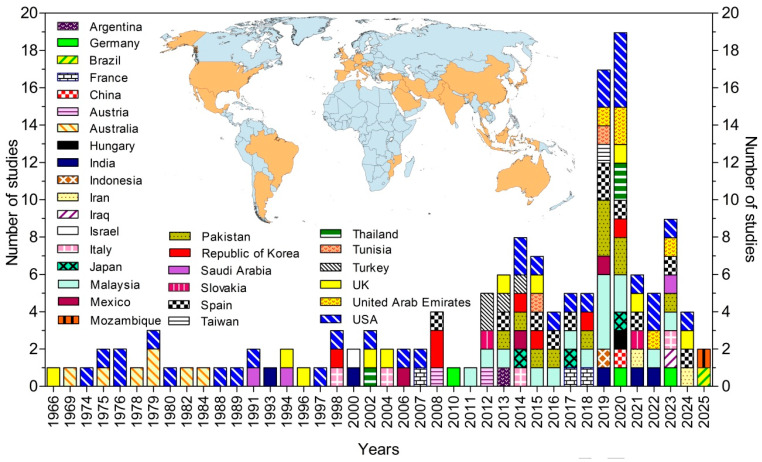
Spatial and temporal distribution of the studies.

Of the 23,624 records retrieved from the databases, 112 studies fully met the predefined inclusion criteria and were therefore included in the final analysis (Figure 1). The included studies were conducted by research groups from 31 countries, representing five continents (Figure 2) [24,25,28,29,30,31,32,33,34,35,36,37,38,39,40,41,42,43,44,45,46,47,48,49,50,51,52,53,54,55,56,57,58,59,60,61,62,63,64,65,66,67,68,69,70,71,72,73,74,75,76,77,78,79,80,81,82,83,84,85,86,87,88,89,90,91,92,93,94,95,96,97,98,99,100,101,102,103,104,105,106,107,108,109,110,111,112,113,114,115,116,117,118,119,120,121,122,123,124,125,126,127,128,129,130,131,132,133,134,135,136,137,138,139].

**Figure 3:**
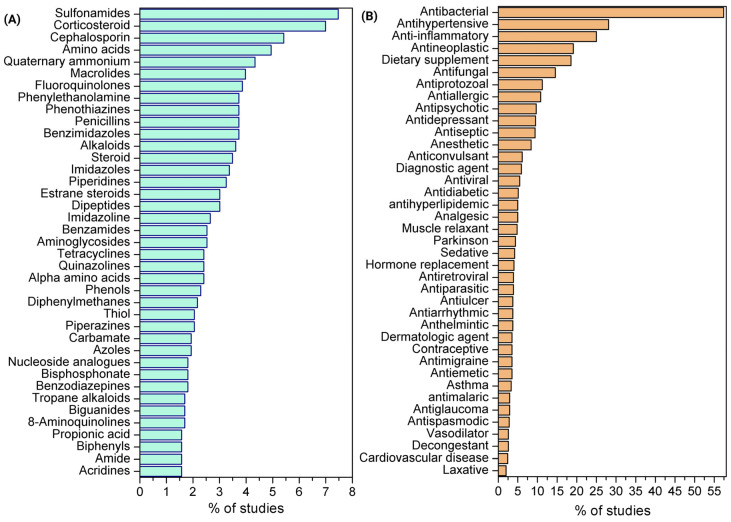
Classes (**A**) and therapeutic indications (**B**) of the most frequently tested drugs.

A total of 2726 drugs and distinct drug combinations were screened for amoebicidal activity using in vitro assays. Among these, 24 compounds (0.9%) were further evaluated in in vivo experimental models, including mice, rats, hamsters, and rabbits. The screened agents belonged to 865 pharmacological classes and had been approved for 565 distinct therapeutic indications. The classification of drugs according to pharmacological class and approved indication is summarized in Figure 3 and detailed in Supplementary Materials.

### 3.1. In Vitro Anti-Free Living Amoeba Activity of Tested Drugs

Among the drugs evaluated in vitro, 329 compounds achieved ≥50% amoebicidal activity when tested across a wide concentration range (0.02–1,450,000 µM). Of these, 54 compounds exhibited amoebicidal activity of comparable magnitude against cyst forms.

Among the drugs displaying ≥50% amoebicidal activity, 221 compounds were active at concentrations ≤ 20 µM, while 166 compounds retained this activity at concentrations ≤ 10 µM, as detailed below.

#### 3.1.1. Drugs with Amoebicidal Activity Against *B. mandrillaris*

**Figure 4:**
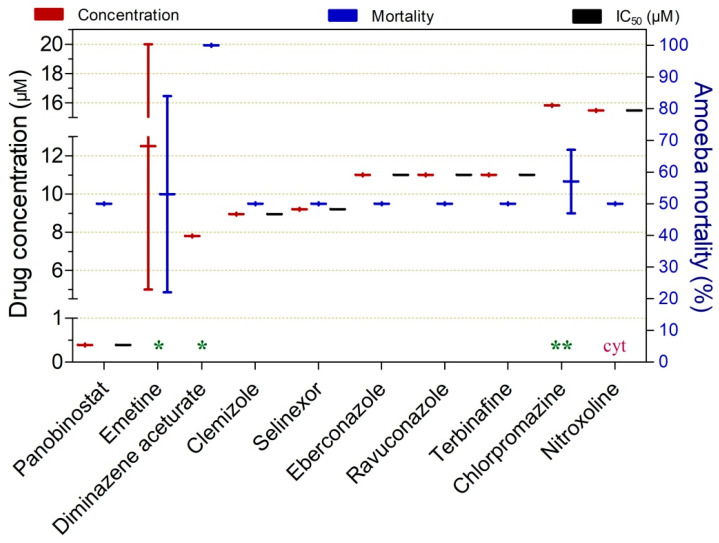
Drugs exhibiting ≥50% in vitro trophocidal activity against *Balamuthia mandrillaris* at concentrations ≤ 15.48 µM. Exposure time was 72 h, except where indicated: (*) 48 h and (**) 120 h. cyt—means cyst and indicates the cysticidal activity of the drug.

Drugs exhibiting substantial balamuthiacidal activity at concentrations ranging from 0.39 to 15.48 µM are presented in Figure 4. Among these, panobinostat was the most potent compound, demonstrating submicromolar activity (IC_50_ = 0.39 µM at 72 h).

Diminazene aceturate (7.8 µM at 48 h; 100% inhibition), clemizole (IC_50_ = 8.95 µM at 72 h), and selinexor (IC_50_ = 9.2 µM at 72 h) also demonstrated notable potency. Nitroxoline was the only compound to exhibit cysticidal activity against *B. mandrillaris*, although this effect was observed at a higher concentration (15.48 µM).

Additional drugs displaying trophocidal activity at a concentration of 20 µM are shown in Figure S1. Among these, nitroxoline stood out for its comparatively higher potency (IC_50_ = 2.8 µM at 72 h).

#### 3.1.2. Drugs with Acanthamoebicidal Activity

Drugs evaluated for acanthamoebicidal activity, including both cysticidal and trophocidal effects, were tested against multiple *Acanthamoeba* species, namely *A. castellanii*, *A. culbertsoni*, *A. griffini*, *A. hatchetti*, *A. palestinensis*, and *A. polyphaga*.

##### Cysticidal Drugs Against *Acanthamoeba* spp.

**Figure 5:**
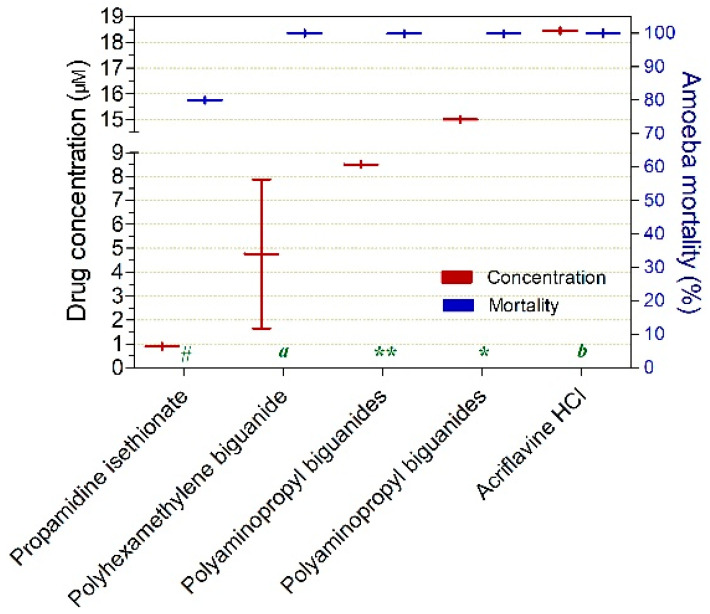
Drugs exhibiting ≥50% in vitro cysticidal activity against *Acanthamoeba* spp. at concentrations ≤20 µM. Exposure times varied as follows: (*) 1 h, (**) 3 h, (#) 4 h, (a) 8 h, and (b) 48 h.

Among the drugs exhibiting cysticidal activity (≥50%) at concentrations ≤ 20 µM, propamidine isethionate (1.0 µM at 4 h; 80%), polyhexamethylene biguanide (8.0 µM at 8 h; 100%), and polyaminopropyl biguanides (8.5 µM at 3 h; 99.9%) demonstrated activity at concentrations ≤ 10 µM (Figure 5).

##### Trophocidal Drugs Against *Acanthamoeba* spp.

**Figure 6:**
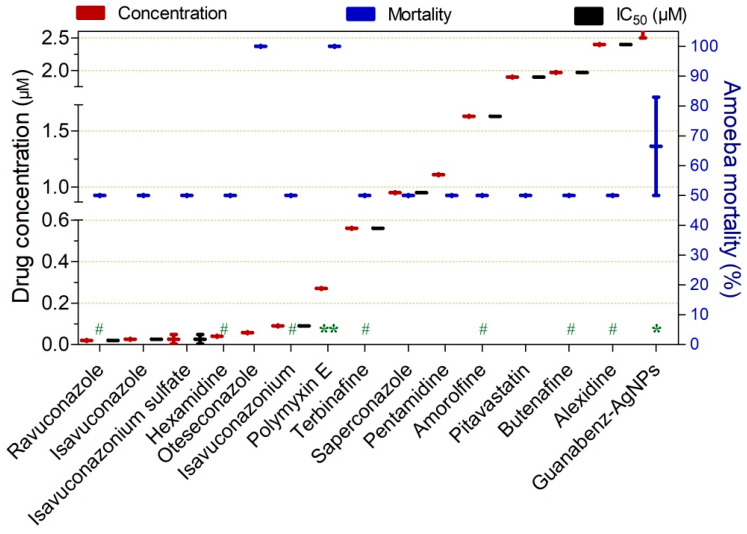
Drugs exhibiting ≥50% trophocidal activity against *Acanthamoeba* spp. at concentrations ranging from 0.02 to 2.5 µM. The exposure time was 48 h, except where indicated: (*) 24 h, (**) 70 h, and (#) 72 h.

Drugs exerting ≥50% trophocidal activity against *Acanthamoeba* spp. at low concentrations (≤2.5 µM) are shown in Figure 6. Among these, ravuconazole (72 h), isavuconazole (48 h), isavuconazonium sulfate (48 h), hexamidine (72 h), oteseconazole (48 h), and isavuconazonium (72 h) demonstrated acanthamoebicidal activity ranging from 50% to 100% at submicromolar concentrations (≤0.2 µM). Polymyxin E (70 h), terbinafine (72 h), and saperconazole (48 h) also exhibited activity at very low concentrations (≤1.0 µM). Notably, oteseconazole, polymyxin E, and guanabenz-AgNPs achieved 100%, 100%, and 83% trophozoite inactivation, respectively (Figure 6).

1. Trophocidal drugs active at intermediate concentrations (3.0 to 5.0 µM)

**Figure 7:**
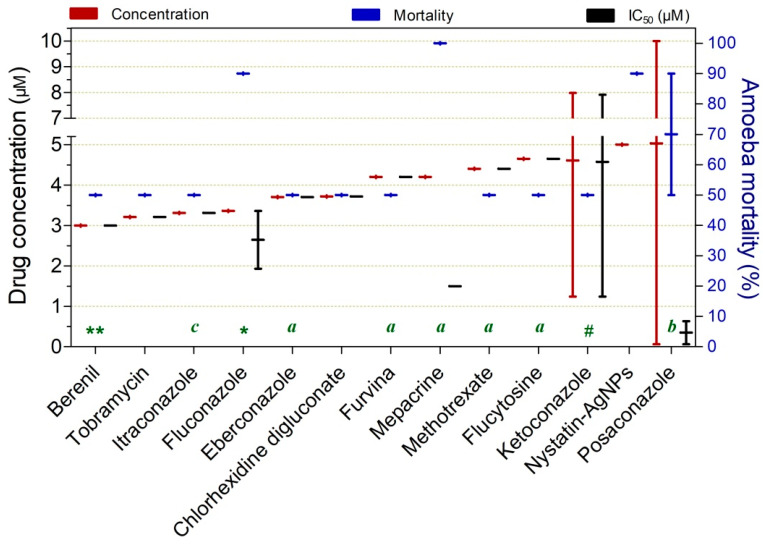
Drugs exhibiting ≥50% trophocidal activity against *Acanthamoeba* spp. at intermediate concentrations ranging from 3.0 to 5.0 µM. The exposure time was 24 h, except where indicated: (*) 12 h, (**) 48 h, (#) 66 h, (a) 72 h, (b) 84 h, and (c) 96 h.

Drugs exerting ≥50% trophocidal activity at intermediate concentrations ranging from 3.0 to 5.0 µM are shown in Figure 7. Among these, fluconazole (3.36 µM at 12 h; 90%; IC_50_ = 1.93 µM), mepacrine (4.2 µM at 72 h; 100%; IC_50_ = 1.5 µM), and nystatin–AgNPs (5.0 µM at 24 h; 90%) demonstrated comparatively higher trophocidal activity at the reported concentrations. Tobramycin (3.21 µM at 24 h; 50%) and berenil (3.21 µM at 24 h; 50%) also stood out by achieving significant activity at shorter exposure times relative to the other compounds.

2. Trophocidal drugs active at higher concentrations 5.6 to 14.6 µM

**Figure 8:**
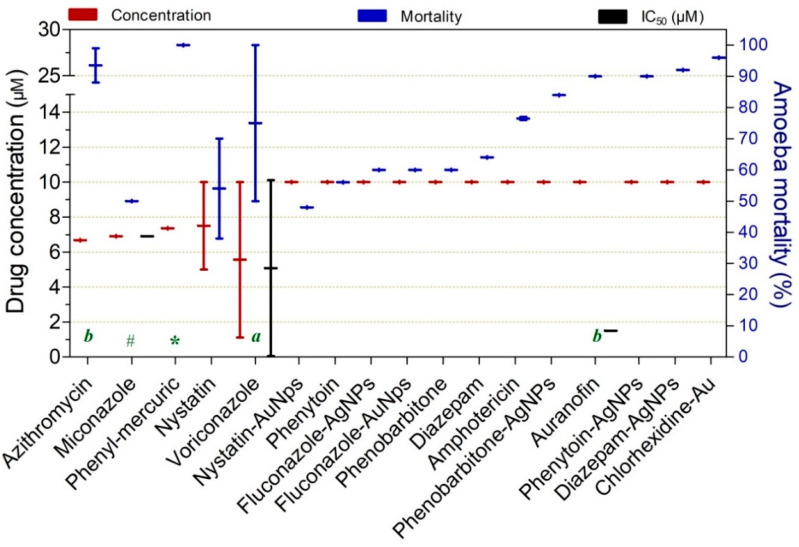
Drugs exhibiting ≥50% trophocidal activity against *Acanthamoeba* spp. at intermediate concentrations ranging from 5.6 to 14.6 µM. The exposure time was 24 h, except where indicated: (*) 4 h, (#) 48 h, (a) 96 h, and (b) 120 h.

Drugs reported to exhibit substantial in vitro trophocidal activity (≥50%) at intermediate-to-higher concentrations ranging from 5.6 to 14.6 µM are shown in Figure 8. Overall, these compounds demonstrated comparable performance; however, miconazole (6.9 µM at 48 h; 50%), azithromycin (6.8 µM at 120 h; 88%), diazepam-AgNPs (10 µM at 24 h; 92%), and auranofin (10 µM at 120 h; 90%; IC_50_ = 1.5 µM) appeared to be relatively more potent within this concentration range.

Compounds exhibiting substantial trophocidal activity at concentrations >10 µM (10–20 µM) are presented in Figure S2. Among these, natamycin (IC_50_ = 2.85 µM at 12 h), chlorhexidine (IC_50_ = 4.28 µM at 96 h), amphotericin B (IC_50_ = 8.65 µM at 72 h), and itraconazole (IC_50_ = 10.1 µM at 96 h) displayed noteworthy IC_50_ values.

##### Drugs Active Against *Naegleria* spp.

1. Cysticidal drugs against *Naegleria* spp.

**Figure 9:**
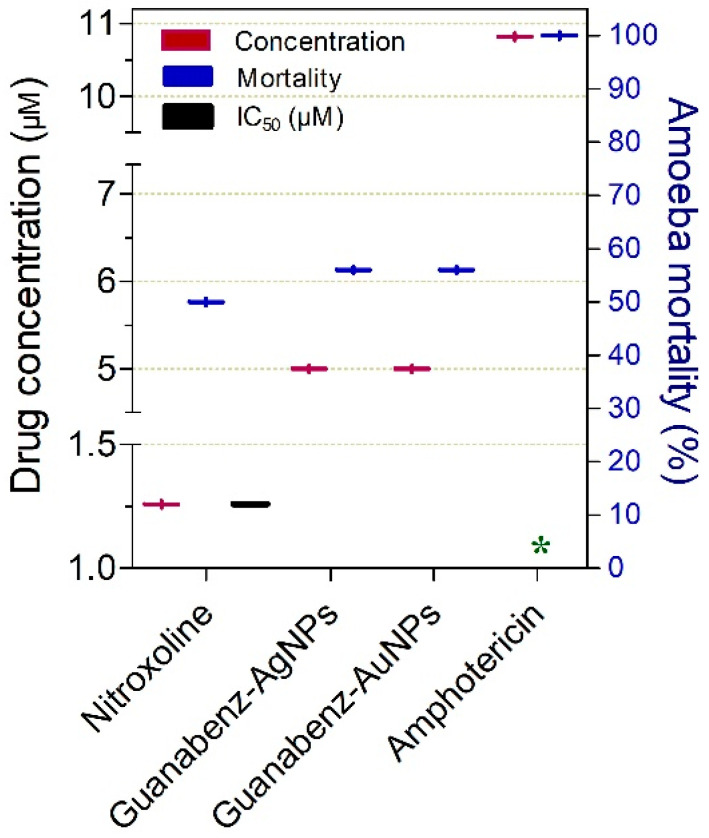
Drugs exhibiting ≥50% cysticidal activity against *Naegleria* spp. at concentrations ranging from 1.26 to 10.82 µM. The exposure time was 24 h, except where indicated: (*) 48 h.

Drugs exhibiting substantial cysticidal activity (≥50%) against *N. fowleri* at clinically relevant concentrations (≤10 µM) are shown in Figure 9. Among these, nitroxoline was the most potent compound, displaying activity at comparatively lower concentrations (IC_50_ = 1.26 µM at 24 h). Nanoformulations of guanabenz, including guanabenz-AgNPs and guanabenz-AuNPs, also demonstrated notable potency (5 µM at 24 h; 56% inhibition). Amphotericin B also exhibited cysticidal activity against *N. fowleri* within this concentration range (Figure 9).

2. Trophocidal drugs against *Naegleria* spp.

Studies evaluating the trophocidal effects of drugs against protist of the genus *Naegleria* were conducted primarily on *N. fowleri* and *N. gruberi*.

**Figure 10:**
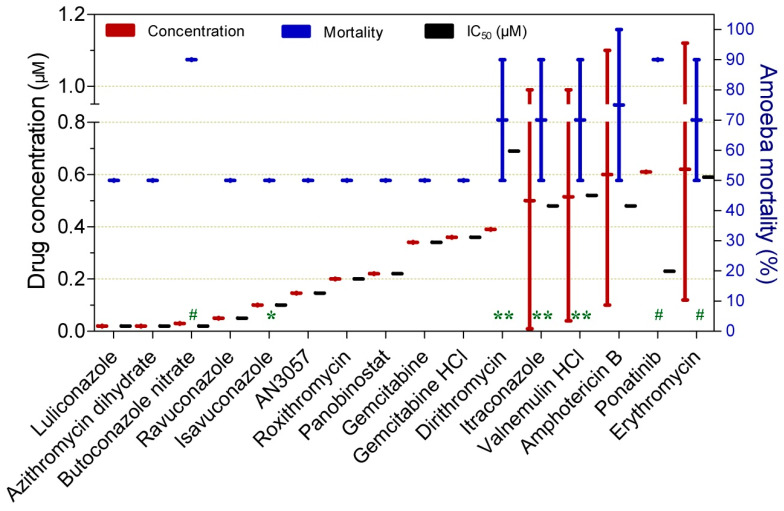
Drugs exhibiting ≥50% trophocidal activity against *Naegleria* spp. at intermediate submicromolar concentrations ranging from 0.02 to 0.62 µM. The exposure time was 72 h, except where indicated: (*) 48 h, (**) 84 h, and (#) 120 h.

Drugs exhibiting substantial trophocidal activity (≥50%) against *Naegleria* spp. at submicromolar concentrations (0.02–0.62 µM) are shown in Figure 10. Although all compounds demonstrated high trophocidal potency, luliconazole, azithromycin dihydrate, butoconazole nitrate, and ravuconazole were particularly notable for achieving this effect at concentrations as low as 0.05 µM. Butoconazole nitrate (0.03 µM at 120 h; 90%; IC_50_ = 0.02 µM), dirithromycin (0.39 µM at 84 h; 90%; IC_50_ = 0.69 µM), itraconazole (0.5 µM at 84 h; 90%; IC_50_ = 0.48 µM), valnemulin HCl (0.52 µM at 84 h; 90%; IC_50_ = 0.52 µM), ponatinib (0.61 µM at 120 h; 90%; IC_50_ = 0.23 µM), and amphotericin B (0.6 µM at 120 h; 90%; IC_50_ = 0.48 µM) were distinguished by achieving higher rates of trophozoite inactivation.

**Figure 11:**
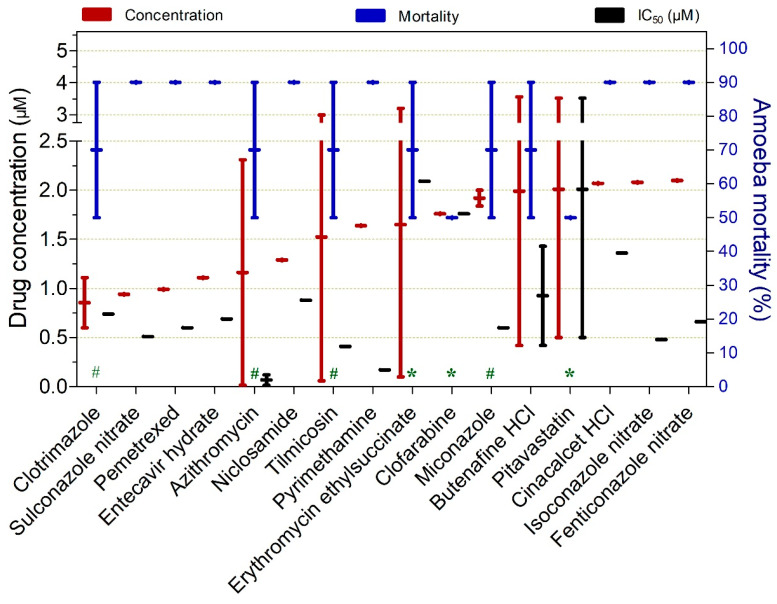
Drugs exhibiting ≥50% trophocidal activity against *Naegleria* spp. at concentrations ranging from 0.86 to 2.1 µM. The exposure time was 120 h, except where indicated: (*) 72 h and (#) 84 h.

Drugs exhibiting substantial trophocidal activity (≥50%) against *Naegleria* spp. at concentrations ranging from 0.86 to 2.1 µM are presented in Figure 11. All compounds demonstrated high and comparable trophocidal potency; however, azithromycin, pyrimethamine, tilmicosin, isoconazole nitrate, sulconazole nitrate, pemetrexed, miconazole, fenticonazole nitrate, entecavir hydrate, clotrimazole, and niclosamide were particularly notable for exhibiting submicromolar IC_50_ values (0.11–0.88 µM).

**Figure 12:**
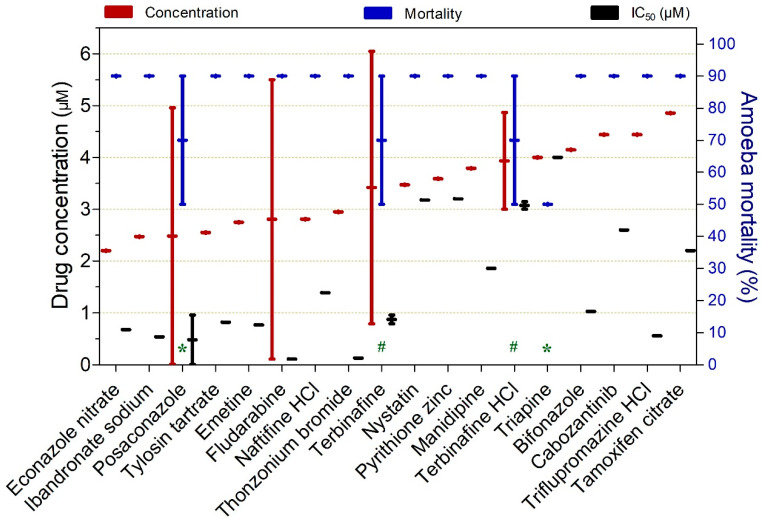
Drugs exhibiting ≥50% trophocidal activity against *Naegleria* spp. at concentrations ranging from 2.2 to 4.9 µM. The exposure time was 120 h, except where indicated: (*) 72 h and (#) 84 h.

Drugs exhibiting ≥ 50% trophocidal activity against *Naegleria* spp. at concentrations ranging from 2.2 to 4.9 µM are presented in Figure 12. Most compounds demonstrated high trophocidal potency, inactivating up to 90% of protist, with the exception of triapine. Among these, fludarabine, thonzonium bromide, ibandronate sodium, triflupromazine HCl, econazole nitrate, emetine, tylosin tartrate, posaconazole, and terbinafine were notable for exhibiting submicromolar IC_50_ values.

**Figure 13:**
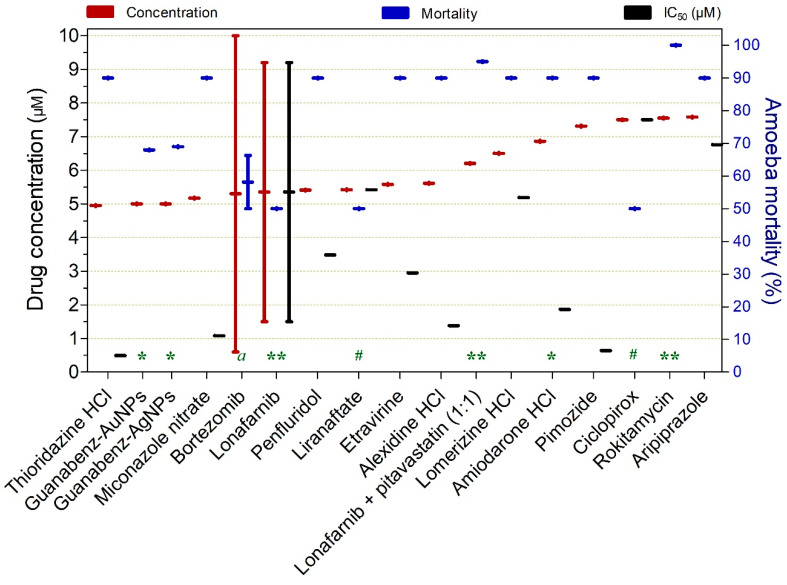
Drugs exhibiting ≥50% trophocidal activity against *Naegleria* spp. at concentrations ranging from 4.95 to 7.58 µM. The exposure time was 120 h, except where indicated: (*) 24 h, (**) 48 h, (#) 72 h, and (a) 84 h.

Drugs exhibiting ≥50% naeglericidal activity against trophozoites at concentrations ranging from 4.95 to 7.58 µM are presented in Figure 13. Overall, all compounds demonstrated high naeglericidal potency; however, the combination of lonafarnib plus pitavastatin (1:1) and rokitamycin were particularly notable for achieving 95% and 100% parasite inactivation, respectively. Thioridazine HCl (IC_50_ = 0.49 µM), pimozide (IC_50_ = 0.64 µM), miconazole nitrate (IC_50_ = 1.08 µM), alexidine HCl (IC_50_ = 1.38 µM), and amiodarone HCl (IC_50_ = 1.86 µM) also stood out by exhibiting IC_50_ values below 2 µM.

**Figure 14:**
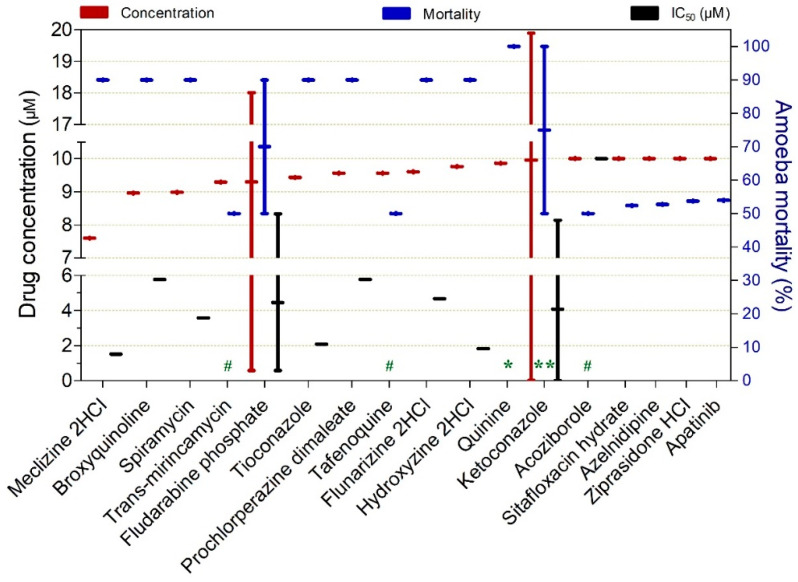
Drugs exhibiting ≥50% trophocidal activity against *Naegleria* spp. at concentrations ranging from 7.6 to 10 µM. The exposure time was 120 h, except where indicated: (*) 48 h, (**) 84 h, and (#) 72 h.

Drugs exhibiting ≥50% trophocidal activity against *Naegleria* spp. at concentrations ranging from 7.6 to 10 µM are presented in Figure 14. Among these, meclizine 2HCl (IC_50_ = 1.52 µM), hydroxyzine 2HCl (IC_50_ = 1.83 µM), tioconazole (IC_50_ = 2.08 µM), spiramycin (IC_50_ = 3.58 µM), flunarizine 2HCl (IC_50_ = 4.67 µM), broxyquinoline (IC_50_ = 5.77 µM), and prochlorperazine dimaleate (IC_50_ = 5.77 µM) were notable for achieving approximately 90% trophozoite inactivation following exposure to concentrations between 7.6 and 9.86 µM for 120 min. Quinine achieved complete trophozoite inactivation (100%) after 48 h of exposure at a concentration of 9.86 µM.

**Figure 15:**
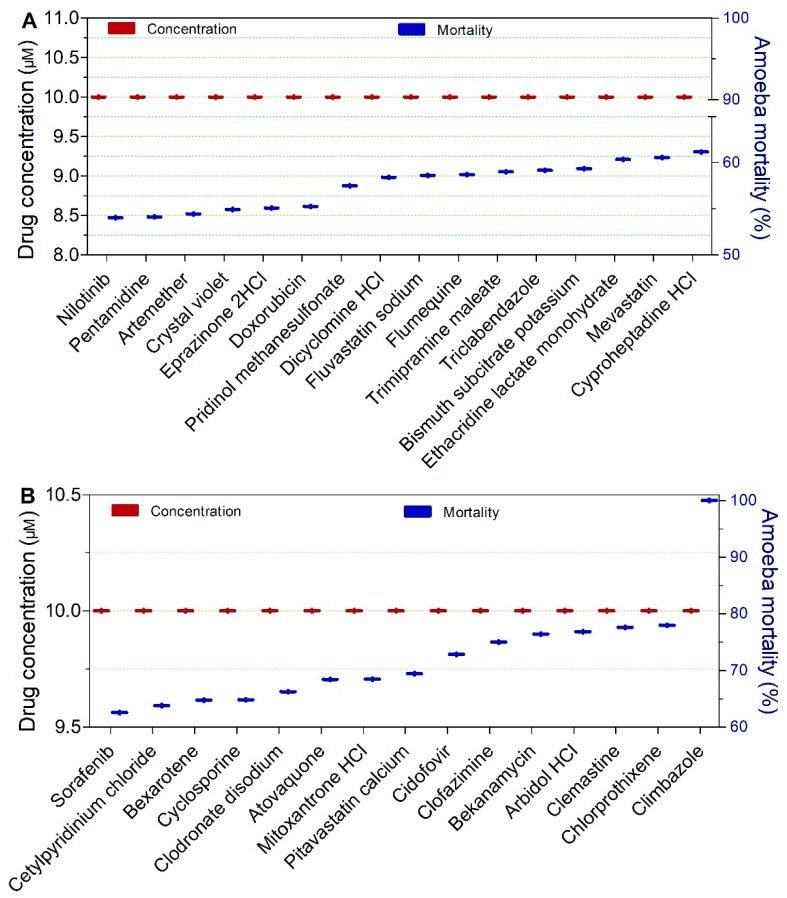
Drugs exhibiting ≥50% trophocidal activity against *Naegleria* spp. at a fixed concentration of 10 µM following 120 h of exposure. (**A**) Drugs with amebicidal activity >60%. (**B**) Drugs with activity >75%.

Drugs exhibiting ≥50% trophocidal activity against *Naegleria* spp. at a fixed concentration of 10 µM and an exposure time of 120 min are presented in Figure 15A,B. Among these, cidofovir, clofazimine, bekanamycin, arbidol HCl, clemastine, and chlorprothixene were notable for achieving trophocidal effects exceeding 70% (72–78%). Climbazole was the most active compound, achieving complete trophozoite inactivation (100%) (Figure 15B). Compounds exhibiting ≥50% trophocidal activity at concentrations > 10 µM are presented in Figures S3 and S4.

### 3.2. In Vivo Performance of Drugs Tested Against FLA Infections

A total of 38 drugs, administered either as monotherapies or in combination, were evaluated for their anti-FLA potential in in vivo models of GAE, PAM, and *Acanthamoeba* keratitis (AK) (Tables S1 and S2).

#### 3.2.1. Therapeutic Performance of Drugs Evaluated In Vivo in Models of Granulomatous Amoebic Encephalitis Caused by *Acanthamoeba*

Among the compounds evaluated in rat models of GAE caused by *Acanthamoeba* spp., sulphadiazine and rifampicin demonstrated the most favorable therapeutic outcomes, achieving cure rates ranging from 88% to 100% (Table 1). More detailed data are presented in Table S1.

#### 3.2.2. In Vivo Efficacy of Drugs Tested in Animal Models of *Acanthamoeba* Keratitis

Drugs evaluated in animal models of AK that achieved cure outcomes in ≥70% of treated animals are summarized in Table 2 and detailed in Table S2. Among these, the combination therapies comprising aprotinin, neomycin, and atropine ointment; neomycin and atropine ointment; polyhexamethylene biguanide, neomycin, and atropine ointment; povidone-iodine, neomycin, and atropine ointment; as well as the combination of miltefosine and polyhexanide, in addition to voriconazole monotherapy, demonstrated the most favorable therapeutic performance, achieving cure rates ranging from 80% to 100%.

Among the drugs evaluated for their repurposing potential to treat PAM in animal models, those achieving cure rates ≥ 55% are presented in Table 3. Notably, the combinations of amphotericin B plus azithromycin (100%) and amphotericin B plus tetracycline (87.5%), as well as the monotherapies cyclophosphamide (92%) and rokitamycin (80%), stood out for achieving the highest cure rates.

## 4. Discussion

Diseases caused by FLA represent a serious and persistent public health challenge and are almost invariably associated with devastating or fatal infections. Despite decades of research aimed at developing new drugs from synthetic compounds and phytoderivatives, there is still no specifically approved treatment for these infections [18,19].

This review critically synthesizes evidence from the major indexing databases on drugs already approved for other clinical indications that have been evaluated for anti-FLA activity, with the objective of identifying the most promising candidates for therapeutic repurposing in the treatment of FLA infections.

Investing in drug-repurposing research for FLA diseases is a rational, cost-effective, and highly promising strategy. This approach is particularly relevant for amoebic encephalitis, where the combination of extreme clinical severity, rarity of cases, frequent diagnostic delay, and rapid symptom progression (often culminating in accelerated clinical deterioration) renders randomized controlled clinical trials largely impracticable.

Drug repurposing leverages established data on biological activity, safety, pharmacokinetics, and pharmacodynamics, integrating these with evidence generated in in vitro, ex vivo, and in vivo models. This framework supports a more efficient, translational assessment of therapeutic potential against FLA, while substantially reducing the time, cost, and risk typically associated with de novo antimicrobial development.

Our findings indicate that, although important knowledge gaps remain, substantial progress has been made. In total, 2726 compounds spanning 865 pharmacological classes and 565 approved therapeutic indications were evaluated for anti-FLA activity. Collectively, these studies identified 166 promising drugs that demonstrated potent in vitro anti-FLA activity at concentrations considered highly translational (≤10 µM), supported by favorable ADMET predictions [140].

### 4.1. In Vitro Studies

Among the promising drugs with anti-*B. mandrillaris* activity (Figure 4), four exhibited greater translational potential (Table 4), demonstrating significant trophocidal activity at concentrations below 10 µM. Notably, none of the evaluated drugs showed cysticidal activity.

*B. mandrillaris* is the etiological agent of GAE, a condition that is rarely diagnosed but associated with an extremely high mortality rate, estimated at approximately 98% [141]. Although cases of oral infection following ingestion of contaminated food have been reported [142], the predominant routes of infection are believed to involve inhalation of airborne cysts or direct entry of the protozoan through lesions in the skin and mucous membranes, including the nasopharyngeal mucosa, as well as transmission via organ transplantation. Following entry into the host, the parasite disseminates hematogenously until it reaches the central nervous system (CNS), [141,143]. Infection has been described in both immunocompetent individuals and those who are immunodeficient or immunosuppressed [144,145,146].

To date, no effective, standardized treatment has been established for *B. mandrillaris* infections. Reported clinical cases are managed empirically with combination regimens that commonly include pentamidine, miltefosine, azithromycin, azole antifungals, and various antibacterial agents [143]. Notably, none of the drugs most frequently cited in case reports overlap with those identified in the present study as candidates with the highest translational potential. It should be emphasized, however, that all drugs listed in Table 4 were evaluated in only a single study, which substantially limits the robustness of the available evidence. Consequently, further investigations—particularly confirmatory in vitro studies and in vivo validation—are essential to substantiate the true therapeutic potential of these candidates.

Among all drugs tested in vitro against *Acanthamoeba* spp., 41 trophocidal agents and three cysticidal agents demonstrated activity at concentrations considered translational (0.02–10 µM; Table 5).

Among all drugs tested in vitro against *Acanthamoeba* spp., 41 trophocidal agents and three cysticidal agents showed activity at concentrations considered translational (0.02–10 µM; Table 5).

*Acanthamoeba* spp. can cause a severe ocular infection, *Acanthamoeba* keratitis, which, although rare (2.34 cases per million eyes; [147]), can lead to irreversible visual loss [148,149]. In addition, this protist is responsible for granulomatous amoebic encephalitis (GAE), a severe CNS infection associated with mortality rates exceeding 90% [16].

Although clinical studies indicate that topical therapies based on polyhexamethylene biguanide (PHMB; 0.02–0.08%) and chlorhexidine (0.02%) achieve high cure rates (78–87% and 86%, respectively) [150,151], there is still no universally effective and approved standard treatment. Management of confirmed cases remains largely empirical and is primarily based on combination regimens that include anti-amoebic agents, such as PHMB, chlorhexidine (0.02–0.06%), propamidine isethionate (Brolene), other diamidines, hexamidine (0.1%), and desomedine (0.1%), often combined with antibacterial, antifungal, and antiviral drugs [152].

Similarly, for systemic *Acanthamoeba* infections, treatment remains empirical and generally relies on pharmacological combinations that may include pentamidine, sulfadiazine, flucytosine, fluconazole, azithromycin, and miltefosine, as well as nitroxoline, voriconazole, isavuconazole, posaconazole, itraconazole, plicamycin, and ponatinib [16].

Drugs approved as antifungals predominate among trophocides active against *Acanthamoeba*, particularly those in the azole class (Table 5). The broad activity of azoles against *Acanthamoeba* spp. is likely related to their inhibition of ergosterol biosynthesis [153]. Ergosterol is one of the major membrane sterols in this protist [154] and appears to be essential for amoebal proliferation and encystment [155]. Notably, the azoles ravuconazole, isavuconazole, isavuconazonium, oteseconazole, and saperconazole showed activity at submicromolar concentrations; however, all except isavuconazonium sulfate were evaluated in only one study, underscoring the need for confirmatory in vitro, ex vivo, and in vivo investigations.

It is also noteworthy that oteseconazole and polymyxin E inactivated 100% of trophozoites at submicromolar concentrations after 48 h and 70 h of exposure, respectively. Mepacrine, fluconazole, azithromycin, chlorhexidine-Au, diazepam-AgNP, auranofin, and phenytoin-AgNPs likewise stood out for high anti-*Acanthamoeba* potency against trophozoites, albeit at relatively higher concentrations.

Regarding cysticidal activity, only antiseptics demonstrated effects at concentrations below 10 µM. Among these, PHMB stood out because of both efficacy and a comparatively favorable toxicity profile. Available data indicate that PHMB is well tolerated when administered as eye drops in healthy individuals (12 times daily for 7 days, followed by six times daily for a further 7 days). A concentration of 0.02% is generally considered safe for therapeutic use, whereas 0.08% may be clinically justifiable in selected therapeutic contexts [100,151].

Among the drugs that exerted a substantial naeglericidal effect (≥IC_50_) at concentrations ≤ 20 µM, a total of 115 trophocidal agents and three cysticidal agents demonstrated activity at potentially translational concentrations (≤10 µM) and are listed in Table 6. *N. fowleri*, a thermophilic and cosmopolitan protist, is the etiological agent of PAM, a disease characterized by extremely rapid progression and an estimated mortality rate of approximately 98%. Moreover, most of the rare survivors present permanent neurological sequelae [156].

The trophocidal drugs identified with anti-*Naegleria* spp. activity at concentrations ≤10 µM predominantly belong to the antifungal class (particularly azoles) and antibacterial agents (mainly macrolides), followed by antineoplastic drugs (e.g., nucleosides and benzanilides), antiprotozoals, and antipsychotics.

As observed for *Acanthamoeba* spp., the consistent activity of azoles against *N. fowleri* is biologically plausible and may be explained by the central role of sterols in the parasite membrane, together with the susceptibility of sterol biosynthesis pathways to pharmacological inhibition by azoles [155]. A similar mechanistic rationale has been proposed for the antineoplastic agent tamoxifen citrate, whose anti-*Naegleria* activity has been associated with disruption of sterol-dependent processes and/or membrane homeostasis [157].

Although additional studies are warranted, posaconazole, ketoconazole, and amphotericin B stand out among antifungals because the literature provides comparatively consistent evidence supporting their promising naeglericidal activity. In contrast, the naeglericidal effects of other antifungal agents remain less well characterized and require stronger bibliographic support, including further experimental validation.

Macrolides (e.g., azithromycin, clarithromycin, and erythromycin), as well as related derivatives (roxithromycin, dirithromycin, spiramycin, tilmicosin/tylosin, and rokitamicin), are antibiotics classically known to inhibit bacterial protein synthesis by binding to the peptidyltransferase region of the 50S ribosomal subunit [158,159]. Although macrolides are traditionally described as inhibitors of bacterial translation, their activity against *N. fowleri* implies the presence of a susceptible target distinct from the eukaryotic cytosolic ribosome. Mechanistic evidence from helminth studies suggests that macrolides may act on the mitochondrial ribosome, thereby impairing translation of essential proteins within the parasite respiratory chain [160]. Given that *N. fowleri* possesses functional mitochondria and shows strong metabolic reliance on energy-generating pathways, it is plausible that macrolides exert anti-amoebic effects predominantly through interference with mitochondrial translation. Nonetheless, direct molecular validation of this mechanism in FLA is still required.

With the exception of azithromycin, whose anti-*Naegleria* activity is well documented (Table 6), including in vivo evidence [125,161], most of the identified macrolides still lack confirmatory studies, both in vitro and in vivo, to substantiate their true translational repurposing potential.

Antineoplastic agents with anti-*Naegleria* activity at promising concentrations span multiple pharmacological classes and mechanisms of action, converging on essential and conserved eukaryotic cellular pathways. A first axis involves genomic damage and replicative stress, exemplified by doxorubicin and mitoxantrone, which intercalate into DNA and disrupt topoisomerase II, ultimately inducing genomic lesions incompatible with cell proliferation [162,163,164]. A second axis relates to nucleotide depletion and inhibition of DNA synthesis. Gemcitabine combines incorporation into DNA with inhibition of ribonucleotide reductase, whereas fludarabine and pemetrexed interfere with DNA polymerases and folate-dependent enzymes, thereby restricting DNA/RNA synthesis; triapine further reinforces this axis by inhibiting ribonucleotide reductase via interaction with its metal center/radical [165,166,167,168].

Disruption of proteostasis represents another critical vulnerability: bortezomib promotes the accumulation of misfolded proteins by inhibiting the 26S proteasome, while panobinostat induces transcriptional and cell cycle dysregulation through HDAC inhibition [169,170,171]. In addition, several kinase inhibitors (ponatinib, nilotinib, sorafenib, cabozantinib, and apatinib) were identified, consistent with the structural conservation of kinase catalytic domains and the likely downstream impact on signaling, proliferation, vesicular trafficking, and motility. Sorafenib targets RAF kinases and receptor tyrosine kinases (e.g., VEGFR/PDGFR), nilotinib and ponatinib were developed against BCR-ABL, and cabozantinib and apatinib act on receptor tyrosine kinases such as VEGFR2 [172,173,174,175,176]. Although designed for mammalian targets, some compounds (such as tamoxifen) may also exert non-genomic effects on pathways including PKC/calmodulin and membrane biophysics, which could be relevant even in the absence of classical homologous receptors [177].

Taken together, these drugs converge on central mechanisms, replicative collapse, nucleotide depletion, proteostasis failure, epigenetic dysregulation, and kinase signaling blockade, providing a plausible mechanistic basis for their activity against *Naegleria* spp. and reinforcing the need for additional studies to confirm amoebicidal effects, selectivity, and feasibility of therapeutic repurposing for PAM.

The identification of emetine, quinine, tafenoquine, artemether, pyrimethamine, and atovaquone as amoebicides against *Naegleria* is also biologically plausible, as these drugs target essential and conserved protozoan processes, including protein translation, redox homeostasis, folate/nucleotide metabolism, and mitochondrial bioenergetics. Emetine, a classic antiamoebic agent, inhibits protein synthesis via ribosomal binding. Direct evidence linking this target to amoebicidal activity has been demonstrated in Entamoeba histolytica, and structural studies of protozoan ribosomes further support this rationale [178,179].

Atovaquone provides the most robustly supported mechanistic axis among the identified agents. It acts as a ubiquinone analog and inhibits the mitochondrial bc1 complex (complex III) at the Qo site, leading to collapse of the mitochondrial membrane potential (ΔΨm) and bioenergetic failure. This mechanism is supported by extensive experimental and structural evidence demonstrating bc1 binding [180,181,182]. In protist, inhibition of bc1 also disrupts respiratory chain, dependent pathways, including pyrimidine biosynthesis via dihydroorotate dehydrogenase (DHODH), thereby amplifying antiparasitic activity [180,183].

Consistently, tafenoquine, an 8-aminoquinoline, has been linked to mitochondrial dysfunction and the induction of oxidative and proteotoxic stress in parasites. Experimental studies have shown inhibition of respiration and induction of apoptosis-like cell death, while comprehensive reviews consolidate oxidative stress as a central component of its mechanism of action [184,185,186]. For artemether, the most widely accepted mechanism involves bioactivation of its endoperoxide bridge by iron or heme, generating reactive radicals that cause multitarget macromolecular damage, as demonstrated in *Plasmodium* spp. [187]. Although biological differences exist, this redox-based mechanism may also be relevant in amoebae under conditions of iron availability and active oxidative metabolism.

Pyrimethamine acts as a competitive inhibitor of dihydrofolate reductase (DHFR), blocking regeneration of tetrahydrofolate required for purine and thymidylate synthesis and, consequently, DNA and RNA production [188]. This supports a mechanism of nucleotide depletion and proliferative arrest in *Naegleria*. In contrast, the mechanism of quinine requires greater caution. In Plasmodium, quinoline compounds interfere with heme detoxification and hemozoin formation, increasing free heme levels and oxidative stress [189,190,191]. As *Naegleria* does not share this pathway, quinine is likely to act through alternative mechanisms, such as redox imbalance, membrane or ion perturbation, or interaction with conserved enzymatic targets, which remain to be specifically validated.

Taken together, the available data support three principal mechanistic axes underlying anti-*Naegleria* activity: (i) inhibition of protein translation (emetine), (ii) mitochondrial dysfunction and oxidative stress (atovaquone, tafenoquine, and potentially artemether), and (iii) blockade of folate-dependent nucleotide metabolism (pyrimethamine). Among these agents, quinine remains the most mechanistically uncertain and warrants targeted investigation using assays of ΔΨm, mitochondrial respiration and ATP production, reactive oxygen species generation, and metabolic rescue.

Antipsychotic trophocidal agents active against *Naegleria* spp. (Table 6), including phenothiazines (e.g., thioridazine and prochlorperazine) and other amphiphilic or cationic neuroleptics (e.g., pimozide and ziprasidone), have also been repeatedly associated with antiparasitic activity through non-canonical mechanisms. This provides a plausible pharmacological rationale for their anti-*Naegleria* effects at potentially translational concentrations. Phenothiazines display pleiotropic actions, including mitochondrial dysfunction (ΔΨm dissipation, bioenergetic impairment, and increased oxidative stress) and disruption of Ca^2+^ homeostasis, mechanisms compatible with rapid lethality in unicellular eukaryotes [192]. Furthermore, evidence from protozoan models indicates that calmodulin/Ca^2+^ antagonists (a class that includes prochlorperazine) interfere with essential Ca^2+^-dependent processes and may induce mitochondrial depolarization, supporting the existence of a Ca^2+^/calmodulin–mitochondria axis as a critical vulnerability in amoebae [193,194].

For thioridazine, beyond the mitochondrial signature, mechanistic reviews indicate that phenothiazines can act as “helper compounds” by interfering with efflux systems and membrane physiology, thereby increasing intracellular drug accumulation and promoting cellular dysfunction. By analogy, this mechanism is biologically plausible in *Naegleria* spp. [195,196]. Pimozide, although classically classified as a dopamine D_2_ receptor antagonist, also interacts with Ca^2+^-dependent pathways, including ion channels. Inhibition of this network can impair motility, endocytosis/phagocytosis, and stress-response pathways, which are critical processes in unicellular eukaryotes [197]. Ziprasidone, despite its receptor-dependent pharmacology in mammals, is likely to exert effects in *Naegleria* through off-target mechanisms related to its amphiphilic and cationic properties, with the potential to modulate membrane organization and intracellular signaling. Accordingly, experimental validation of ziprasidone should prioritize phenotypes indicative of bioenergetic collapse and Ca^2+^ imbalance, such as ΔΨm dissipation, ATP depletion, reactive oxygen species generation, and altered Ca^2+^ dynamics, rather than mechanistic inference based on human receptor targets [198,199].

Taken together, a parsimonious mechanistic model for ziprasidone, pimozide, thioridazine, and prochlorperazine in *Naegleria* spp. involves the convergence of three major processes: (1) mitochondrial dysfunction accompanied by oxidative stress, (2) disruption of Ca^2+^ homeostasis and calmodulin-dependent signaling, and (3) perturbation of membrane integrity and transporter function. These mechanisms are well documented for phenothiazines and calmodulin antagonists across diverse microorganisms and align with fundamental vulnerabilities of FLA [196,200].

As widely established in the literature, the cyst form of FLA is markedly less susceptible to drugs than the trophozoite form, largely due to the presence of a double-layered wall enriched in cellulose, β-glucans, chitin, and tectins [5]. Consistent with this, our results (Table 5 and Table 6) show that only 3.62% (6/166) of the drugs that exhibited substantial anti-FLA activity (≥IC_50_) at potentially translational concentrations (≤10 µM) also demonstrated cysticidal activity.

Cyst-inactivating agents are particularly desirable for *Acanthamoeba* infections, including keratitis and granulomatous amoebic encephalitis, because this protozoan can encyst at the infection site itself [149,201]. Use of non-cysticidal drugs may induce encystment, leading to an apparent regression of symptoms while increasing the likelihood of relapse after treatment discontinuation and raising the risk of complications. This is especially relevant in post-surgical contexts, which are frequently required in keratitis management [149,202].

It is also important to note that, although only six drugs showed substantial cysticidal effects at potentially translational concentrations (≤10 µM), a total of 55 drugs displayed relevant cysticidal activity (≥IC_50_). In most cases, however, this activity was observed only at higher concentrations, as widely reported in the literature [88,94,121]. These findings underscore the difficulty of identifying agents with effective cysticidal activity at pharmacologically viable levels. They also emphasize the strategic value of drugs capable of inhibiting trophozoite encystment, which may help optimize therapeutic responses and reduce relapse risk [48].

### 4.2. In Vivo Studies

#### 4.2.1. In Vivo Models for Granulomatous Amoebic Encephalitis

Of the drugs evaluated in vivo against GAE, only sulfadiazine and rifampicin achieved high cure rates in a murine model (Table 1). Sulfadiazine (200 mg/kg, with treatment initiated 1 day post-infection resulted in 100% cure, a finding supported by a well-established antifolate mechanism of action. Sulfonamides act as competitive antagonists of para-aminobenzoic acid (PABA), inhibiting dihydropteroate synthase (DHPS) and thereby disrupting folate biosynthesis, a pathway essential for nucleotide synthesis and cellular proliferation. This mechanism is corroborated by in vitro evidence in *Acanthamoeba* spp., where sulfonamide-mediated growth inhibition is reversed by supplementation with PABA or folic acid, indicating a predominantly on-target effect [89]. Nevertheless, only limited acanthamebicidal activity has been observed in vitro at high drug concentrations [48], despite clinical reports associating sulfadiazine-containing regimens with patient survival in GAE [203]. The therapeutic efficacy appears to be highly dependent on early intervention, in agreement with experimental data demonstrating a marked loss of activity once infection is established within the CNS [59].

Rifampicin also demonstrated high efficacy, achieving complete protection when administered prophylactically, but showing a pronounced reduction in effectiveness when treatment was initiated after infection onset [50]. Notably, rifampicin has been reported to lack direct amebicidal activity in vitro [129], suggesting that its in vivo efficacy may be mediated by indirect or host-dependent mechanisms. Collectively, these findings underscore the critical importance of treatment timing in GAE. However, the current evidence base is limited to a small number of experimental studies, underscoring the need for independent replication and systematic evaluation across later therapeutic windows before translational conclusions can be drawn.

#### 4.2.2. In Vivo Models for *Acanthamoeba* Keratitis

Data summarized in Table 2 indicate that the drug combinations of aprotinin, neomycin, and atropine ointment; neomycin and atropine ointment; PHMB, neomycin, and atropine ointment; and povidone-iodine, neomycin, and atropine ointment achieved 100% cure rates in rabbit models of *Acanthamoeba* keratitis. Notably, neomycin and atropine were present in all curative regimens, suggesting a relevant contribution of these agents to therapeutic efficacy.

Although neomycin is classically described as an inhibitor of bacterial protein synthesis, its anti-*Acanthamoeba* activity is likely mediated by indirect mechanisms. These include inhibition of mitochondrial translation, given the bacterial origin of mitochondrial ribosomes, as well as disruption of plasma membrane integrity and interference with phosphoinositide-dependent signaling pathways [148,204].

Atropine, a classical muscarinic antagonist, has demonstrated antiparasitic activity against other protists, including Cryptosporidium parvum (in vitro and in vivo) [205] and Plasmodium vivax (in vitro) [206]. In *Acanthamoeba*, bioinformatic and structural analyses have suggested the presence of putative muscarinic-like binding sites or distant receptor homologs [207,208]. However, canonical muscarinic receptors comparable to those described in mammals have not been conclusively identified.

Accordingly, the antiparasitic effects of atropine in *Acanthamoeba* are more plausibly mediated through indirect mechanisms, including disruption of intracellular calcium homeostasis and modulation of phosphoinositide-dependent signaling pathways involved in motility, cytoskeletal organization, and encystment [209,210].

Although further studies are required to elucidate the precise molecular targets of neomycin and atropine in *Acanthamoeba* spp., the mechanistic considerations discussed above help to explain the amebostatic activity of neomycin and its historical use as an adjuvant in the management of *Acanthamoeba* keratitis. These effects may also partially account for the high cure rates observed in rabbit models, particularly when neomycin and atropine are combined with established anti-*Acanthamoeba* agents such as PHMB, fluconazole, povidone-iodine, or aprotinin [95,108,150,211].

Notably, PHMB-based regimens, especially those combined with aprotinin, were among the most promising therapeutic approaches. In addition, treatment protocols including PHMB at a concentration of 0.02% have been reported to exhibit improved ocular tolerability, an important consideration for prolonged topical therapy [99].

Further studies are warranted to evaluate these combinations in vivo under conditions that more closely resemble clinical scenarios, including delayed initiation of treatment and more advanced or established infections, to better define their translational and therapeutic potential.

The performance of voriconazole as monotherapy, achieving an 88.9% cure rate even when treatment was initiated 7 days post-infection in murine models, is noteworthy (Table 2). This therapeutic effect is strongly supported by its potent in vitro trophocidal activity against *Acanthamoeba* spp. [62,95], despite the absence of significant cysticidal activity [127,128].

The curative efficacy of voriconazole is primarily attributed to its inhibition of ergosterol biosynthesis, a key component of *Acanthamoeba* membrane integrity and viability [153]. Importantly, these experimental findings are supported by a pilot randomized clinical trial, which demonstrated favorable outcomes with voriconazole therapy in patients with *Acanthamoeba* keratitis [212].

Notably, in vivo studies in murine models have shown that the therapeutic efficacy of voriconazole is markedly higher when administered topically as eye drops (88.9%) compared with oral administration (33.3%) [59]. This difference likely reflects improved local drug exposure at the site of infection and the ability of topical delivery to overcome pharmacokinetic limitations associated with systemic administration. Together, these findings underscore the relevance of topical voriconazole as a key component of therapeutic strategies for *Acanthamoeba* keratitis.

Collectively, these data highlight the strong potential of voriconazole for drug repurposing as monotherapy against *Acanthamoeba* keratitis. However, its limited activity against cysts suggests that combination therapy with a cysticidal or anti-encystment agent may be advantageous to enhance treatment durability and reduce recurrence. This approach warrants further investigation in future preclinical and clinical studies.

Although the combination of miltefosine and polyhexanide achieved a relevant cure rate (80%) in murine models of *Acanthamoeba* keratitis (Table 2), this regimen was also associated with a concerning level of toxicity (69%). This toxicity appears to be largely attributable to polyhexanide, as its administration as monotherapy resulted in a 70% cure rate but was accompanied by 65% toxicity. In contrast, miltefosine monotherapy achieved a comparable cure rate (73%) with minimal toxicity (2%) [105].

The strong anti-*Acanthamoeba* activity of miltefosine is consistently documented against trophozoites [77,88,92] and, at higher concentrations, against cysts [45,119]. Miltefosine exerts its anti-*Acanthamoeba* activity through a multifactorial mechanism, primarily involving disruption of plasma membrane integrity, mitochondrial dysfunction, and induction of apoptosis-like cell death, together with interference in phospholipid-dependent signaling pathways essential for motility, encystment, and cyst viability [213,214,215].

Despite these favorable mechanistic features, the therapeutic performance of miltefosine in the treatment of *Acanthamoeba* keratitis (whether used as monotherapy, as an adjunct, or in combination regimens) remains inconsistent across available studies. Further investigations are therefore required to more precisely define its clinical efficacy, optimize dosing strategies, and identify usage approaches that maximize therapeutic benefit while minimizing toxicity.

The promising curative activity of nitazoxanide (80%), formulated in a liquid crystal system, observed in rabbit models of *Acanthamoeba* keratitis (Table 2), is supported by its moderate trophocidal activity and a pronounced anti-encystment effect. Experimental evidence indicates that nitazoxanide interferes with mitochondrial metabolism in *A. castellanii*, impairing anaerobic energy production despite the organism’s metabolic flexibility [216].

Nitazoxanide classically inhibits the pyruvate: ferredoxin oxidoreductase (PFOR)-dependent electron transfer system, a key pathway in protozoan energy metabolism [217]. The recent identification of a functional PFOR enzyme in *A. castellanii* cysts, where it plays a central role in cyst energy homeostasis, provides a strong mechanistic basis for the observed anti-encystment activity and suggests potential impairment of cyst viability [218].

The discrepancy between the modest in vitro trophocidal activity and favorable in vivo efficacy [118,216], suggests that nitazoxanide’s therapeutic benefit in AK may primarily derive from inhibition of encystment and metabolic destabilization of cysts, rather than rapid trophozoite killing. Additionally, the liquid crystal formulation likely enhances corneal penetration and local bioavailability, further contributing to its therapeutic performance. Collectively, these features support nitazoxanide as a mechanistically rational candidate for repositioning in AK, particularly as a topical agent or in combination with fast-acting trophocidal drugs.

#### 4.2.3. In Vivo Models of Primary Amoebic Meningoencephalitis

Our findings (Table 3) show that among the drugs evaluated for repositioning in murine models of PAM, the highest cure rates were achieved with amphotericin B plus azithromycin (100%), azithromycin monotherapy (100%), cyclophosphamide (92%), amphotericin B plus tetracycline (87.5%), and rokitamycin (80%). The therapeutic success of these regimens reflects a combination of direct anti-*Naegleria* activity and indirect host-mediated mechanisms, including modulation of inflammatory responses and enhanced drug penetration into the CNS.

Amphotericin B remains the cornerstone of PAM therapy due to its direct amoebicidal activity [44], mediated by high-affinity binding to ergosterol-like sterols in the *Naegleria* plasma membrane, resulting in pore formation, ion leakage, and rapid cell death [219,220]. Its efficacy against *N. fowleri* trophozoites has been consistently demonstrated both in vitro and in vivo [161,221].

The addition of azithromycin likely provides a synergistic effect, combining membrane disruption with inhibition of mitochondrial and plastid-like ribosomal protein synthesis [124,161]. Azithromycin has been shown to accumulate intracellularly and penetrate the CNS, where it may impair *Naegleria* mitochondrial translation and metabolic activity [222,223]. Moreover, azithromycin exerts anti-inflammatory and immunomodulatory effects [224], potentially mitigating host-mediated neuronal damage during PAM. Together, these complementary mechanisms plausibly account for the complete protection observed in murine models.

The observation that azithromycin alone achieved 100% cure is particularly noteworthy, as it suggests a potent direct anti-*Naegleria* effect independent of amphotericin B. Mechanistically, azithromycin inhibits protein synthesis by binding to the 50S ribosomal subunit [225], and in protists, this effect is thought to preferentially target mitochondrial ribosomes, which retain bacterial ancestry [226].

In *N. fowleri*, mitochondrial function is critical for energy production, motility, and thermotolerance [227]. Disruption of mitochondrial translation may therefore lead to metabolic collapse and trophozoite death. In addition, azithromycin’s favorable pharmacokinetic profile, including high tissue penetration and prolonged intracellular retention [222], likely contributes to sustained amoebicidal exposure within the brain parenchyma. These features position azithromycin as a particularly attractive candidate for PAM therapy and warrant further mechanistic and translational investigation.

Unlike classical anti-amoebic agents, cyclophosphamide does not appear to exert direct antiparasitic activity against *Naegleria*. Rather, the therapeutic benefit observed in murine PAM models is most plausibly attributable to a host-directed effect. Cyclophosphamide is a well-established immunosuppressive and anti-inflammatory agent [228], with documented capacity to reduce leukocyte infiltration, pro-inflammatory cytokine production, and cerebral edema [229]. In PAM, disease progression and mortality are influenced not only by parasite burden but also by severe neuroinflammatory responses [230]. Accordingly, modulation of the host inflammatory response may contribute to improved outcomes by limiting intracranial hypertension, preserving neuronal integrity, and indirectly facilitating parasite control. These observations underscore the relevance of immunopathology as a therapeutic target in PAM; however, the clinical translatability of cyclophosphamide remains constrained by its toxicity profile [231] and the risk of systemic immunosuppression.

The combination of amphotericin B and tetracycline demonstrated substantial efficacy in murine PAM models, supporting the potential value of a dual-target therapeutic strategy. Amphotericin B exerts direct amoebicidal activity through disruption of *Naegleria* membrane integrity [220], whereas tetracycline inhibits protein synthesis via binding to the 30S ribosomal subunit [232], a mechanism that may impair mitochondrial translation [233].

Although tetracyclines generally exhibit weaker direct amoebicidal activity than macrolides, their interference with mitochondrial metabolism, together with their anti-inflammatory properties [234], may contribute to therapeutic benefit when used in combination with amphotericin B. The slightly lower cure rate observed relative to the amphotericin B–azithromycin regimen may be related to pharmacokinetic differences, including comparatively lower CNS penetration and intracellular accumulation. Collectively, these findings support combination approaches targeting complementary parasite and host pathways in PAM, while highlighting variability in efficacy across adjunctive agents.

Rokitamycin, a 16-membered macrolide, achieved an 80% cure rate in murine models of PAM (Table 3), further supporting the relevance of macrolides as candidate agents against *N. fowleri*. Although direct mechanistic data in *Naegleria* are unavailable, its activity is consistent with the class mechanism of macrolides, which inhibit protein synthesis via binding to the 50S ribosomal subunit [225], an effect that, in protozoa, is thought to preferentially impair mitochondrial ribosomes [226,233]. This interpretation is supported by earlier in vitro evidence of amoebostatic and amoebicidal activity of rokitamycin against *A. castellanii*, as well as synergistic effects when combined with amphotericin B or chlorpromazine [87]. Given the critical role of mitochondrial function in *N. fowleri* pathogenicity [227], disruption of mitochondrial translation represents a biologically plausible contributor to its in vivo efficacy. The comparatively lower cure rate relative to azithromycin may reflect differences in pharmacokinetics, including tissue distribution and CNS penetration, which are known to vary across macrolides. These findings reinforce the concept that macrolides constitute a promising and still underexplored class of anti-*Naegleria* agents, particularly as components of combination regimens.

Collectively, these findings suggest that the most effective regimens against PAM tend to combine agents with direct amoebicidal activity (e.g., amphotericin B) with drugs that may impair mitochondrial function, inhibit protein synthesis, or modulate host-driven immunopathology. Macrolides, in particular, appear to be promising candidates for drug repositioning, given their antiparasitic activity, immunomodulatory properties, favorable pharmacokinetic profiles, and capacity to penetrate the CNS. Taken together, these observations support further translational investigation of repositioned drugs, combination regimens, and host-directed therapeutic strategies to improve outcomes in this otherwise highly lethal disease.

Certain methodological aspects may limit the interpretation of the findings of the present study, including the adoption of a potency-based screening threshold (IC_50_ ≤ 10 μM), which, although necessary to ensure methodological consistency and comparability across studies, may underrepresent drugs that have demonstrated clinical application despite modest in vitro activity. Different agents have been incorporated into therapeutic regimens for FLA, including encephalitis, largely based on compassionate use and combination therapy rather than exclusively on robust pharmacological potency data [156]. Accordingly, the exclusion of these drugs from the highest-priority categories in this review should not be interpreted as a lack of therapeutic relevance, but rather as a consequence of the predefined experimental criteria systematically applied. Furthermore, it should be considered that the inclusion or exclusion of several compounds among the most promising candidates was, in multiple instances, based on evidence derived from a single study available in the literature, sometimes generated using only a single experimental strain, which limits the robustness and generalizability of the conclusions.

## 5. Conclusions

To synthesize the current state of progress in research on potentially repurposable drugs for the treatment of free-living amoeba infections, a systematic and critical review of the scientific literature was conducted, encompassing both in vitro and in vivo studies. This review evaluated drugs tested in vitro against *B. mandrillaris*, *Acanthamoeba* spp., and *Naegleria* spp., as well as those assessed in in vivo models of GAE, AK, and PAM.

Out of a total of 2726 drugs evaluated, 166 compounds tested in vitro exhibited potent amoebicidal activity (≥IC_50_) at potentially translatable concentrations (≤10 µM).

Among the most promising in vitro agents against *B. mandrillaris* trophozoites, panobinostat and diminazene aceturate stood out, showing activity at concentrations of 0.39 and 7.8 µM, respectively.

Against *Acanthamoeba* spp., the most promising trophocidal agents included ravuconazole, isavuconazole, isavuconazonium sulfate, hexamidine, oteseconazole, polymyxin E, terbinafine, and saperconazole, all of which demonstrated activity at submicromolar concentrations (0.02–0.95 µM). Additional compounds—including pentamidine, amorolfine, pitavastatin, butenafine, alexidine, mepacrine, azithromycin, fluconazole, voriconazole, chlorhexidine-Au, diazepam-AgNPs, auranofin, and phenytoin-AgNPs—also ranked among the most promising trophocidal agents due to their activity at low micromolar concentrations (1.1–2.4 µM) or their high biocidal effect (≥90%). Notably, only propamidine isethionate, polyhexamethylene biguanide, and polyaminopropyl biguanides exhibited cysticidal activity against *Acanthamoeba* spp.

Among the drugs active against *Naegleria* spp., several compounds demonstrated submicromolar potency (0.002–0.99 µM), including luliconazole, azithromycin dihydrate, butoconazole nitrate, ravuconazole, isavuconazole, AN3057, roxithromycin, panobinostat, gemcitabine, gemcitabine HCl, dirithromycin, itraconazole, valnemulin HCl, ponatinib, clarithromycin, erythromycin, clotrimazole, sulconazole nitrate, and pemetrexed. Only nitroxoline, guanabenz-AgNPs, and guanabenz-AuNPs exhibited cysticidal activity against *Naegleria*, with efficacy at promising concentrations (1.3–5 µM).

In vivo evidence for GAE remains limited and highly dependent on treatment timing. Only sulfadiazine and rifampicin demonstrated high efficacy in experimental models, primarily when administered early or prophylactically, underscoring the critical importance of early intervention and the need for independent validation across later therapeutic windows before translational relevance can be established.

In in vivo models of AK, PHMB-based regimens combined with aprotinin plus neomycin achieved high therapeutic efficacy with improved ocular tolerability. Voriconazole demonstrated robust efficacy as topical monotherapy, even when treatment was initiated at later stages; however, its limited cysticidal activity suggests that combination with anti-encystment agents may be required. Nitazoxanide, formulated in a liquid crystal delivery system, also showed promising in vivo activity, although it was associated with some degree of toxicity.

Finally, in vivo models of PAM identified amphotericin B-azithromycin combination therapy, as well as azithromycin monotherapy, as the most promising therapeutic strategies, reinforcing the potential of macrolide-based regimens for drug repositioning in this highly lethal infection.

## Figures and Tables

**Table 1:** Comparative performance of drugs evaluated in rat models of granulomatous amoebic encephalitis (GAE) caused by *Acanthamoeba* spp. All compounds were administered via subcutaneous injection, except where indicated (*) for intraperitoneal administration.

Drug	Studies (n)	Dose (mg/kg)	Treatment Time (days)	Start of Treatment dpi	Cure Rate (%)
Sulphadiazine *	1	200	10	1	100
Rifampicin	1	100	2	2 **	100
Rifampicin	1	100	5	2	88

(dpi) Days post-infection. (**) Before infection (prophylactic treatment).

**Table 2:** Drugs demonstrating the highest cure rates in animal models of *Acanthamoeba* keratitis. Studies were conducted in mice, hamsters (*), and rabbits (**).

Drug (mg/mL)	Administration Route	Dose (mg/mL)	Treatment Time (days)	Start of Treatment dpi	Cure Rate (%) ±SD	Toxicity (%)
Aprotinin + neomycin (0.283) + Atropine ointment (10)	Eye drops **	13	17 (5×/day)	2	100	-
Fluconazole + neomycin (0.283) + Atropine ointment (10)	Eye drops **	2	22 (5×/day)	2	100	-
Neomycin (0.283) + atropine ointment (10)	Eye drops **	13	28 (5×/day)	2	100	-
PHMB + neomycin (0.283) + atropine ointment (10)	Eye drops **	0.2	13 (5×/day)	2	100	-
Povidone iodine + neomycin (0.283) + Atropine ointment (10)	Eye drops **	50	23 (5×/day)	2	100	-
Nitazoxanide (in liquid crystal)	Eye drops **	10	30 (5×/day)	3	80 ± 0.0	20–70
Voriconazole	Eye drops	10	21 (13×/day/3 days; 7×/day/11 days and 4×/day/7 days)	7	88.9 ± 0.0	-
Miltefosine + polyhexanide	Eye drops	0.06512 + 0.2	28 (8×/day/1st wk; 3×/day/2 wks)	5	80 ± 0.0	69
Miltefosine	Eye drops *	0.06512	29 ± 1 (8×/day/1st wk; 3×/day/3 wks)	5	73 ± 13	2
Chlorhexidine + neomycin sulfate + polymyxin B sulfate + gramicidin	Eye drops	0.02 + 4.49 + 0.5 + 0.025	7 (8×/day) + 21 (3×/day)	5	71 ± 5	
Miltefosine + propamidine isethionate	Eye drops	0.06512 + 0.2	28 (8×/day/1st wk; 3×/day/2 wks)	5	70 ± 0.0	79
Miltefosine + chlorhexidine	Eye drops	0.06512 + 0.2	28 (8×/day/1st wk; 3×/day/2 wks)	5	70 ± 0.0	77
Polyhexanide	Eye drops	0.2	28 (8×/day/1st wk; 3×/day/2 wks)	5	70 ± 0.0	65

(dpi) Days post-infection. PHMB—Polyhexamethylene biguanide.

**Table 3:** Performance of drugs tested in mouse models of primary amoebic meningoencephalitis caused by *N. fowleri*. All drugs were administered intraperitoneally.

Drug	Studies (n)	Dose (mg/kg)	Treatment Time (Days)	Start of Treatment dpi	Cure Rate (%)	Toxicity
Amphotericin B + azithromycin	1	2.5 + 25	5	3	100	-
Cyclophosphamide	1	30	10	0	92	-
Amphotericin B + tetracycline	1	2.5 + 150	7	3	87.5	-
Rokitamycin	1	20	3 (3×/day) *	3	80	No
Chlorpromazine	1	20	3 *	3	75	-
Azithromycin + posaconazole	1	25 + 20	3	3	70	-
Amphotericin B	1	7.5	14	1≥	60	-
Azithromycin	1	25	5	3	55	
Azithromycin	1	75	5	3	100	
Miltefosine	1	20	3 *	3	55	-

(dpi) Days post-infection. (*) Animals were treated on days 3, 7 and 11 after infection. (-) Unreported data.

**Table 4:** Most promising drugs identified in vitro against *Balamuthia mandrillaris* trophozoites. Concentration values represent the mean and corresponding ± SD.

Drug	Drug Class	Approved for	Studies (n)	Concentration (µM), ± SD	Mortality (%)	IC_50_ (µM), ± SD	Exposure Time (h)
Panobinostat	Histone deacetylase inhibitor	Cancer	1	0.39 ± 0.0	50	0.39 ± 0.0	72
Diminazene aceturate	Diamidines	Antiprotozoal	1	7.8 ± 0.0	100		48
Clemizole	Benzimidazoles	Allergic rhinitis, urticaria	1	8.95 ± 0.0	50	8.95 ± 0.0	72
Selinexor	Triazoles	Cancer	1	9.2 ± 0.0	50	9.2 ± 0.0	72

SD—Standard deviation. Empty cells indicate unavailable data.

**Table 5:** Most promising drugs identified in vitro against *Acanthamoeba* spp. Concentration values represent the mean and corresponding ± SD.

Drug	Drug Class	Approved for	Studies (n)	Concentration ((SD), µM)	Mortality (%), ±SD	IC_50_ (µM), ±SD	Exposure Time (h)
**Trophozoites**							
Ravuconazole	Azoles	Antifungal	1	0.02 ± 0.0	50 ± 0.0	0.02 ± 0.0	72
Isavuconazole	Azoles	Antifungal	1	0.03 ± 0.0	50 ± 0.0	0.026 ± 0.0	48
Isavuconazonium sulfate	Azoles	Antifungal	3	0.03 ± 0.03	50 ± 0.0	0.03 ± 0.03	48
Hexamidine	Hexamidine	Disinfectant	1	0.04 ± 0.0	50 ± 0.0		72
Oteseconazole	Azoles	Antifungal	1	0.06 ± 0.0	100		48
Isavuconazonium	Azoles	Antifungal	1	0.09 ± 0.0	50 ± 0.0	0.09 ± 0.0	72
Polymyxin E	Cationic polypeptide	Antibacterial	1	0.27 ± 0.0	100		70
Terbinafine	Allylamine	Antifungal	1	0.56 ± 0.0	50 ± 0.0	0.56 ± 0.0	72
Saperconazole	Azoles	Antifungal	1	0.95 ± 0.0	50 ± 0.0	0.95 ± 0.0	48
Pentamidine	Diamidines	Disinfectant, antiseptic	1	1.11 ± 0.0	50 ± 0.0		72
Amorolfine	Morpholine derivative	Antifungal	1	1.63 ± 0.0	50 ± 0.0	1.63 ± 0.0	72
Pitavastatin	Statins	Heart disorders	1	1.90 ± 0.0	50 ± 0.0	1.9 ± 0.0	48
Butenafine	Naftifine	Antifungal	1	1.97 ± 0.0	50 ± 0.0	1.97 ± 0.0	72
Alexidine	BisBiguanides	Antibacterial	1	2.40 ± 0.0	50 ± 0.0	2.4 ± 0.0	72
Guanabenz-AgNPs	Alpha-2 adrenérgicos	Antihypertensive	1	3.75 ± 1.77	66.5 ± 23.3	5 ± 0.0	24
Berenil	Diamidines	Antiprotozoal	1	3.00 ± 0.0	50 ± 0.0	3 ± 0.0	48
Tobramycin	Aminoglycoside	Antibacterial	1	3.21 ± 0.0	50 ± 0.0	3.21 ± 0.0	24
Itraconazole	Azoles	Antifungal	1	3.31 ± 0.0	50 ± 0.0	3.31 ± 0.0	96
Fluconazole	Azoles	Antifungal	5	3.36 ± 0.0	90 ± 0.0	2.7 (1.01)	12
Eberconazole	Azoles	Antifungal	1	3.70 ± 0.0	50 ± 0.0	3.7 ± 0.0	72
Chlorhexidine digluconate	Biguanides	Antiseptic	1	3.72 ± 0.0	50 ± 0.0	3.72 ± 0.0	96
Furvina	Nitrofurans	Antibacterial, Antifungal	1	4.20 ± 0.0	50 ± 0.0	4.2 ± 0.0	72
Mepacrine	Acridines	Antiprotozoal	1	4.20 ± 0.0	100 ± 0.0	1.5 ± 0.0	72
Methotrexate	Antimetabolites	Antineoplastic	1	4.40 ± 0.0	50 ± 0.0	4.4 ± 0.0	72
Flucytosine	Pyrimidine analogues	Antifungal	1	4.65 ± 0.0	50 ± 0.0	4.65 ± 0.0	72
Ketoconazole	Azoles	Antifungal	5	4.61 ± 4.77	50 ± 0.0	4.6 ± 4.72	66
Posaconazole	Azoles	Antifungal	2	5.03 ± 7.03	70 ± 28.3	0.35 ± 0.4	84
Azithromycin	Macrolide	Antibacterial	13	6.68 ± 0.0	94 ± 7.8		120
Miconazole	Azoles	Antifungal	1	6.90 ± 0.0	50 ± 0.0	6.9 ± 0.0	50
Phenyl-mercuric nitrate	Organomercurials	Antiseptic	1	7.36 ± 0.0	100 ± 0.0		4
Voriconazole	Azoles	Antifungal	4	5.56 ± 6.3	75 ± 35.4	5.1 ± 7.1	96
Chlorhexidine-Au	Biguanides	Antiseptic	1	10 ± 0.0	96 ± 0.0		24
Diazepam-AgNP	Benzodiazepine	Antiseizure	1	10 ± 0.0	92 ± 0.0		24
Auranofin	Gold derivative	Antirheumatic	1	10 ± 0.0	90 ± 0.0	1.5 ± 0.0	120
Phenytoin-AgNPs	Hydantoin derivative	Antiseizure	1	10 ± 0.0	90 ± 0.0		24
Phenobarbitone-AgNPs	Barbiturate	Antiepileptic	1	10 ± 0.0	84 ± 0.0		24
Amphotericin B-AuNps	Polyenes	Antifungal	2	10 ± 0.0	76 ± 0.0		24
Diazepam	Benzodiazepine	Antiseizure	1	10 ± 0.0	64 ± 0.0		24
Fluconazole-AuNps	Azoles	Antifungal	1	10 ± 0.0	60 ± 0.0		24
Phenobarbitone	Barbiturate	Antiepileptic	1	10 ± 0.0	60 ± 0.0		24
Phenytoin	Hydantoin derivative	Antiseizure	1	10 ± 0.0	56 ± 0.0		24
**Cysts**							
Propamidine isethionate	Aromatic diamidines	Antimicrobial/Antiseptic	1	0.89 ± 0.0	80 ± 0.0		4
Polyhexamethylene biguanide	Biguanides	Disinfectant, antiseptic	2	1.64 ± 0.0	100 ± 0.0		18 ± 14
Polyaminopropyl biguanides	Biguanides	Antiseptic	1	8.5 ± 0.0	99.9 ± 0.0		3

SD—Standard deviation. Empty cells indicate unavailable data.

**Table 6:** Most active and promising drugs identified in vitro against *Naegleria* spp. Concentration values represent the mean and corresponding ± SD.

Drug	Drug Class	Approved for	Studies (n)	Concentration (µM) ± SD,	Mortality (%) ± SD	IC_50_ (µM) ± SD	Exposure Time (h)
**Trophozoites**							
Luliconazole	Azoles	Antifungal	1	0.02 ± 0.0	50 ± 0.0	0.02 ± 0.0	72
Azithromycin dihydrate	Macrolide	Antibacterial	1	0.02 ± 0.0	50 ± 0.0	0.02 ± 0.0	72
Butoconazole nitrate	Azoles	Antifungal	1	0.03 ± 0.0	90 ± 0.0	0.02 ± 0.0	120
Ravuconazole	Azoles	Antifungal	1	0.05 ± 0.0	50 ± 0.0	0.05 ± 0.0	72
Isavuconazole	Azoles	Antifungal	1	0.1 ± 0.0	50 ± 0.0	0.1 ± 0.0	48
AN3057	Boronics	Antifungal	1	0.146 ± 0.0	50 ± 0.0	0.146 ± 0.0	72
Roxithromycin	Macrolide	Antibacterial	1	0.2 ± 0.0	50 ± 0.0	0.2 ± 0.0	72
Panobinostat	Histone deacetylase inhibitor	Antineoplastic	1	0.22 ± 0.0	50 ± 0.0	0.22 ± 0.0	72
Gemcitabine	Nucleosides	Antineoplastic	1	0.34 ± 0.0	50 ± 0.0	0.34 ± 0.0	72
Gemcitabine HCl	Nucleosides	Antineoplastic	1	0.36 ± 0.0	50 ± 0.0	0.36 ± 0.0	72
Dirithromycin	Macrolide	Antibacterial	2	0.39 ± 0.0	70 ± 28.3	0.69 ± 0.0	84
Itraconazole	Azoles	Antifungal	2	0.5 ± 0.69	70 ± 28.3	0.48 ± 0.0	84
Valnemulin HCl	Pleuromutilins	Antibacterial	2	0.515 ± 0.67	70 ± 28.3	0.52 ± 0.0	84
Ponatinib	Benzanilides	Antineoplastic	1	0.61 ± 0.0	90	0.23 ± 0.0	120
Clarithromycin	Macrolide	Antibacterial	1	0.61 ± 0.82	70 ± 28.3	0.45 ± 0.6	72
Erythromycin	Macrolide	Antibacterial	1	0.62 ± 0.71	70 ± 28.3	0.59 ± 0.0	120
Clotrimazole	Azoles	Antifungal	2	0.855 ± 0.36	70 ± 28.3	0.74 ± 0.0	84
Sulconazole nitrate	Azoles	Antifungal	1	0.94 ± 0.0	90 ± 0.0	0.51 ± 0.0	120
Pemetrexed	Antimetabolites	Antineoplastic	1	0.99 ± 0.0	90 ± 0.0	0.6 ± 0.0	120
Entecavir hydrate	Nucleoside analogues	Antiviral	1	1.11 ± 0.0	90 ± 0.0	0.69 ± 0.0	120
Azithromycin	Macrolide	Antibacterial	12	1.16 ± 1.62	70 ± 28.3	0.07 ± 0.1	84
Niclosamide	Salicylanilide	Anthelmintic	1	1.29 ± 0.0	90 ± 0.0	0.88 ± 0.0	120
Tilmicosin	Macrolide	Antibacterial	2	1.525 ± 2.07	70 ± 28.3	0.41 ± 0.0	84
Pyrimethamine	Pyrimedines	Antiprotozoal	1	1.64 ± 0.0	90 ± 0.0	0.17 ± 0.0	120
Erythromycin ethylsuccinate	Macrolide	Antibacterial	1	1.65 (2.19)	70 ± 28.3	2.09 ± 0.0	72
Clofarabine	Nucleosides	Cancer	1	1.76 ± 0.0	50 ± 0.0	1.76 ± 0.0	72
Miconazole	Azoles	Antifungal	1	1.92 ± 0.11	70 ± 28.3	0.6 ± 0.0	84
Butenafine HCl	Naftifine	Antifungal	2	1.99 ± 2.22	70 ± 28.3	0.93 ± 0.7	120
Pitavastatin	Statins	Heart disorders	1	2.01 ± 2.14	50 ± 0.0	2.01 ± 2.1	72
Cinacalcet HCl	Naphthalenes	Hypercalcemia	1	2.07 ± 0.0	90 ± 0.0	1.36 ± 0.0	120
Isoconazole nitrate	Azoles	Antifungal	1	2.08 ± 0.0	90 ± 0.0	0.48 ± 0.0	120
Fenticonazole nitrate	Azoles	Antifungal	1	2.1 ± 0.0	90 ± 0.0	0.66 ± 0.0	120
Econazole nitrate	Azoles	Antifungal	1	2.2 ± 0.0	90 ± 0.0	0.68 ± 0.0	120
Ibandronate sodium	Bisphosphonate	Osteoporosis	1	2.47 ± 0.0	90	0.54 ± 0.0	120
Posaconazole	Azoles	Antifungal	13	2.48 ± 3.5	70 ± 28.3	0.48 ± 0.7	72
Tylosin tartrate	Macrolide	Antibacterial	1	2.55 ± 0.0	90 ± 0.0	0.82 ± 0.0	120
Emetine	Alkaloids	Antiprotozoal	1	2.75 ± 0.0	90 ± 0.0	0.77 ± 0.0	120
Fludarabine	Nucleosides	Antineoplastic	2	2.81 ± 3.81	90 ± 0.0	0.11 ± 0.0	120
Thonzonium bromide	Quaternary ammonium	Antiseptic	1	2.95 ± 0.0	90 ± 0.0	0.13 ± 0.0	120
Terbinafine	Allylamine	Antifungal	1	3.42 ± 3.72	70 ± 28.3	0.88 ± 0.1	84
Pyrithione zinc	Zinc complex	Antifungal	1	3.59 ± 0.0	90 ± 0.0	3.2 ± 0.0	120
Manidipine	Dihydropyridine	Antihypertensive	1	3.79 ± 0.0	90 ± 0.0	1.86 ± 0.0	120
Terbinafine HCl	Allylamine	Antifungal	2	3.94 ± 1.32	70 ± 28.3	3.08 ± 0.1	84
Triapine	Thiosemicarbazone derivative	Antineoplastic	1	4 ± 0.0	50 ± 0.0	4 ± 0.0	72
Bifonazole	Azoles	Antifungal	1	4.15 ± 0.0	90 ± 0.0	1.03 ± 0.0	120
Cabozantinib	Quinidines	Antineoplastic	1	4.44 ± 0.0	90 ± 0.0	2.6 ± 0.0	120
Triflupromazine HCl	Phenothiazines	Psychiatric disorders	1	4.44 ± 0.0	90 ± 0.0	0.56 ± 0.0	120
Tamoxifen citrate	Triphenylethylene	Antineoplastic	1	4.86 ± 0.0	90 ± 0.0	2.2 ± 0.0	120
Thioridazine HCl	Phenothiazines	Antipsychotic	1	4.95 ± 0.0	90 ± 0.0	0.49 ± 0.0	120
Guanabenz-AuNPs	Alpha-2 adrenérgicos	Antihypertensive	1	5 ± 0.0	68 ± 0.0		24
Guanabenz-AgNPs	Alpha-2 adrenérgicos	Antihypertensive	1	5 ± 0.0	69 ± 0.0		24
Miconazole nitrate	Azoles	Antifungal	1	5.17 ± 0.0	90 ± 0.0	1.08 ± 0.0	120
Bortezomib	Proteasome inhibitor	Antineoplastic	2	5.3 ± 6.7	58 ± 11.5		84
Lonafarnib	Benzocycloheptapyridine	Hutchinson-Gilford syndrome	5	5.4 ± 5.4	50 ± 0.0	5.4 ± 5.4	48
Penfluridol	Diphenylbutylpiperidines	Antiseptic	1	5.41 ± 0.0	90 ± 0.0	3.48 ± 0.0	120
Amphotericin B	Polyenes	Antifungal	3	5.41 ± 5.0	85 ± 15	0.48 ± 0.0	48
Liranaftate	Naftifine	Antifungal	1	5.42 ± 0.0	50 ± 0.0	5.42 ± 0.0	72
Etravirine	Diarylethers	Antivirals (antiretro)	1	5.58 ± 0.0	90 ± 0.0	2.95 ± 0.0	120
Alexidine HCl	BisBiguanides	Antibacterial	1	5.61 ± 0.0	90 ± 0.0	1.38 ± 0.0	120
Lonafarnib + pitavastatin (1:1)			1	6.2 ± 0.0	95 ± 0.0		48
Lomerizine HCl	Piperazines	Antimigraine, antivertigo	1	6.5 ± 0.0	90 ± 0.0	5.19 ± 0.0	120
Amiodarone HCl	Benzofurans	Antiarrhythmic	1	6.86 ± 0.0	90 ± 0.0	1.86 ± 0.0	120
Pimozide	Diphenylbutylpiperidine	Antipsychotic	1	7.31 ± 0.0	90 ± 0.0	0.64 ± 0.0	120
Ciclopirox	Pyridine derivatives	Antifungal	1	7.5 ± 0.0	50 ± 0.0	7.5 ± 0.0	72
Rokitamycin	Macrolide	Antibacterial	1	7.55 ± 0.0	100 ± 0.0		48
Aripiprazole	Azoles	Antifungal	1	7.58 ± 0.0	90 ± 0.0	6.76 ± 0.0	120
Meclizine 2HCl	Piperazines	Antiallergic	1	7.6 ± 0.0	90 ± 0.0	1.52 ± 0.0	120
Broxyquinoline	Haloquinolines	Fungicides, antiprotozoal	1	8.96 ± 0.0	90 ± 0.0	5.77 ± 0.0	120
Spiramycin	Macrolide	Antibacterial	1	8.98 ± 0.0	90 ± 0.0	3.58 ± 0.0	120
Trans-mirincamycin	Lincosamide	Antibacterial	1	9.29 ± 0.0	50 ± 0.0	9.29 ± 0.0	72
Fludarabine phosphate	Nucleosides	Antineoplastic	2	9.3 ± 12.3	70 ± 28.3	4.46 ± 5.5	84
Tioconazole	Azoles	Antifungal	1	9.43 ± 0.0	90 ± 0.0	2.08 ± 0.0	120
Prochlorperazine dimaleate	Phenothiazines	Antipsychotic	1	9.56 ± 0.0	90 ± 0.0	5.77 ± 0.0	120
Tafenoquine	Aminoquinoline	Antiprotozoal	1	9.56 ± 0.0	50 ± 0.0	9.56 ± 0.0	72
Flunarizine 2HCl	Diphenylpiperazine	Antimigraine, antivertigo	1	9.6 ± 0.0	90 ± 0.0	4.67 ± 0.0	120
Hydroxyzine 2HCl	Piperazines	Antiallergic	1	9.76 ± 0.0	90 ± 0.0	1.83 ± 0.0	120
Quinine	Alkaloids	Antiprotozoal	1	9.86 ± 0.0	100 ± 0.0		48
Ketoconazole	Azoles	Antifungal	15	10 ± 14	75 ± 35.4	4.08 ± 5.7	84
Climbazole	Azoles	Antifungal	1	10 ± 0.0	100 ± 0.0		120
Chlorprothixene	Phenothiazines	Psychiatric disorders	1	10 ± 0.0	78 ± 0.0		120
Clemastine	Benzyl ethers	Antihistamine	1	10 ± 0.0	78 ± 0.0		120
Arbidol HCl	Phenylthiazoles	Antiviral	1	10 ± 0.0	77 ± 0.0		120
Bekanamycin	Aminoglycosides	Antibacterial	1	10 ± 0.0	76 ± 0.0		120
Clofazimine	Phenazine	Antibacterial	1	10 ± 0.0	75 ± 0.0		120
Cidofovir	Pyrimidines	Antiviral	1	10 ± 0.0	73 ± 0.0		120
Pitavastatin calcium	Statins	Heart disorders	1	10 ± 0.0	69 ± 0.0		120
Atovaquone	Quinones	Antiprotozoal	1	10 ± 0.0	68 ± 0.0		120
Mitoxantrone HCl	Anthracenedione	Antineoplastic	1	10 ± 0.0	68 ± 0.0		120
Clodronate disodium	Bisphosphonate	Osteoporosis, hypercalcemia	1	10 ± 0.0	66 ± 0.0		120
Bexarotene	Retinoid	Antineoplastic	1	10 ± 0.0	65 ± 0.0		120
Cyclosporine	Cyclic lipopeptides	Immunosuppressant	1	10 ± 0.0	65 ± 0.0		120
Cetylpyridinium chloride	Pyridinium	Antiseptic	1	10 ± 0.0	64 ± 0.0		120
Sorafenib	Diarylethers	Antineoplastic	1	10 ± 0.0	63 ± 0.0		120
Cyproheptadine HCl	Piperidines	Antiallergic	1	10 ± 0.0	61 ± 0.0		120
Mevastatin	Delta valerolactones	Antihyperlipidemic	1	10 ± 0.0	61 ± 0.0		120
Ethacridine lactate monohydrate	Acridines	Antiseptic, abortifacient	1	10 ± 0.0	60 ± 0.0		120
Bismuth subcitrate potassium	Tricarboxylic acid	Antiulcer	1	10 ± 0.0	59 ± 0.0		120
Flumequine	Fluoroquinolones	Antibacterial	1	10 ± 0.0	59 ± 0.0		120
Fluvastatin sodium	Statins	Antihyperlipidemic	1	10 ± 0.0	59 ± 0.0		120
Triclabendazole	Azoles	Antifungal	1	10 ± 0.0	59 ± 0.0		120
Trimipramine maleate	Tricyclic	Antidepressant	1	10 ± 0.0	59 ± 0.0		120
Dicyclomine HCl	Carboxylic acid ester	Antispasmodic	1	10 ± 0.0	58 ± 0.0		120
Pridinol methanesulfonate	Piperidines	Muscle relaxant	1	10 ± 0.0	57 ± 0.0		120
Crystal violet	Triphenylmethane dye	Antibacterial, antifungal	1	10 ± 0.0	55 ± 0.0		120
Doxorubicin	Anthracycline	Antineoplastic	1	10 ± 0.0	55 ± 0.0		120
Eprazinone 2HCl	Benzomorphan	Antitussive	1	10 ± 0.0	55 ± 0.0		120
Apatinib	Quinazolines	Antineoplastic	1	10 ± 0.0	54 ± 0.0		120
Artemether	Artemisinins	Antiprotozoal	1	10 ± 0.0	54 ± 0.0		120
Nilotinib	Benzanilides	Antineoplastic	1	10 ± 0.0	54 ± 0.0		120
Pentamidine	Diamidines	Disinfectant, antiseptic	1	10 ± 0.0	54 ± 0.0		120
Ziprasidone HCl	Benzisothiazole	Antipsychotic	1	10 ± 0.0	54 ± 0.0		120
Azelnidipine	Dihydropyridine derivative	Heart disorders	1	10 ± 0.0	53 ± 0.0		120
Sitafloxacin hydrate	Fluoroquinolone	Antibacterial	1	10 ± 0.0	52 ± 0.0		120
Acoziborole	Azoles	Antifungal	1	10 ± 0.0	50 ± 0.0	10 ± 0.0	72
**Cysts**							
Nitroxoline	Quinoline derivative	Antibacterial	1	1.26 ± 0.0	50 ± 0.0	1.26 ± 0.0	24
Guanabenz-AgNPs	Alpha-2 adrenérgicos	Antihypertensive	1	5 ± 0.0	56 ± 0.0		24
Guanabenz-AuNPs	Alpha-2 adrenérgicos	Antihypertensive	1	5 ± 0.0	56 ± 0.0		24

SD—Standard deviation. Empty cells indicate unavailable data.

## Data Availability

The raw data supporting the conclusions of this article will be made available by the authors upon request.

## Supplementary Materials

The following supporting information can be downloaded at https://www.mdpi.com/article/10.3390/pathogens15030294/s1, Figure S1: Drugs exhibiting ≥50% trophocidal activity against *Balamuthia mandrillaris* at average concentrations of 20 µM; Figure S2: Drugs exhibiting ≥50% trophocidal activity against *Acanthamoeba* spp. at mean concentrations ranging from 10.1 to 23.06 µM; Figure S3: Drugs exhibiting ≥50% trophocidal activity against *Naegleria* spp. at mean concentrations ranging from 10 to 12.89 µM; Figure S4: Drugs that exert a trophocidal effect ≥50% against *Naegleria* spp. at average concentrations ranging from 13 to 19.09 µM. Table S1: Comparative performance of drugs tested in rat models of Granulomatous Amebic Encephalitis caused by *Acanthamoeba* spp. Table S2: Therapeutic efficacy of drugs against *Acanthamoeba* keratitis in animal models; Table S3: Drug efficacy in mouse models of primary amoebic meningoencephalitis caused by *Naegleria fowleri*; Table S4: Complete list of drugs evaluated against free-living amoebae in the literature. File S1. PRISMA_2020_checklist.

## Abbreviations

The following abbreviations are used in this manuscript:

ADMET	Absorption, Distribution, Metabolism, Excretion, and Toxicity
AK	Acanthamoeba keratitis
CNS	Central Nervous System
DHFR	Dihydrofolate reductase
DHODH	Dihydroorotate dehydrogenase
DHPS	Dihydropteroate synthase
dpi	Days post-infection
FLA	Free-Living Amoebae
GAE	granulomatous amoebic encephalitis
HDAC	Histone deacetylases
IC_50_	Inhibitory concentration of 50% of the parasites
PABA	Para-aminobenzoic acid
PAM	Primary Amoebic Meningoencephalitis
PDGFR	Platelet-Derived Growth Factor Receptor
PHMB	Polyhexamethylene Biguanide
PRISMA	Preferred Reporting Items for Systematic Reviews and Meta-Analyses
SD	Standard deviation.
VEGFR	Vascular Endothelial Growth Factor Receptor
ΔΨm	Mitochondrial membrane potential
