# Supplementary material for: Promising Drug Repurposing Candidates Targeting Free-Living Amoebae: A Systematic and Critical Review of Laboratory-Based Evidence
Source: Pathogens. 2026 Mar 7;15(3):294. doi: 10.3390/pathogens15030294 (PMC13028894; doi:10.3390/pathogens15030294)
Supplement: Supplementary file 1 [file pathogens-15-00294-s001.zip › Supplementary material.pdf]

# Supplementary material

*Review*

# Promising Drug Repurposing Candidates Targeting Free-Living Amoebae: A Systematic and Critical Review of Laboratory-Based Evidence

**Beni Jequicene Mussengue Chauque 1,2,3,* , Luiza Bernardes Chagas 4 , Thaisla Cristiane Borella da Silva 5, Denise Leal dos Santos 1, Luciano Palmeiro Rodrigues 1,6 , Lucile da Silva Lins Baia 2, Manoella Kessler Gomes Rodrigues 7, Guilherme Brittes Benitez 8 , Thais Lemos Mendes 2, Hellen Kempfer Philippsen 9 , Luciana Dalla Rosa 10, Fabricio Souza Campos 11,12 , Marilise Brittes Rott 5, Regis Adriel Zanette 2 and Jose Roberto Goldim 1**

1 Master's Program in Clinical Research, Hospital de Clinicas de Porto Alegre, Porto Alegre 90035-903, RS, Brazil
2 Postgraduate Program in Biological Sciences—Pharmacology and Therapeutics, Federal University of Rio Grande do Sul, Porto Alegre 90010-150, RS, Brazil
3 Center of Studies in Science and Technology (NECET), Biology Course, Universidade Rovuma, Lichinga P.O. Box 04, Niassa, Mozambique
4 Faculty of Pharmacy, Federal University of Rio Grande do Sul, Porto Alegre 90010-150, RS, Brazil
5 Protozoology Laboratory, Microbiology Immunology and Parasitology Department, Basic Health Sciences Institute, Federal University of Rio Grande do Sul, Porto Alegre 90010-150, RS, Brazil
6 Physiotherapy Course, Federal University of Rio Grande do Sul, Porto Alegre 90010-150, RS, Brazil
7 School of Medicine, Pontificia Universidade Catolica do Rio Grande do Sul (PUCRS), Porto Alegre 90610-970, RS, Brazil
8 Industrial and Systems Engineering Graduate Program, Polytechnic School, Pontifical Catholic University of Parana (PUCPR), Curitiba 80215-901, PR, Brazil
9 Socio-Environmental and Water Resources Institute, Federal Rural University of the Amazon (UFRA), Belem 66077-830, PA, Brazil
10 Central Laboratory for Avian Disease Diagnosis, Department of Preventive Veterinary Medicine, Center for Rural Sciences, Universidade Federal de Santa Maria, Santa Maria 97105-900, RS, Brazil
11 Laboratorio de Bioinformatica & Biotecnologia, Instituto de Ciências Basicas da Saude, Federal University of Rio Grande do Sul, Porto Alegre 90010-150, RS, Brazil
12 Department of Public & Ecosystem Health, College of Veterinary Medicine, Cornell University, Ithaca, NY 14853, USA
* Correspondence: benichauq@gmail.com

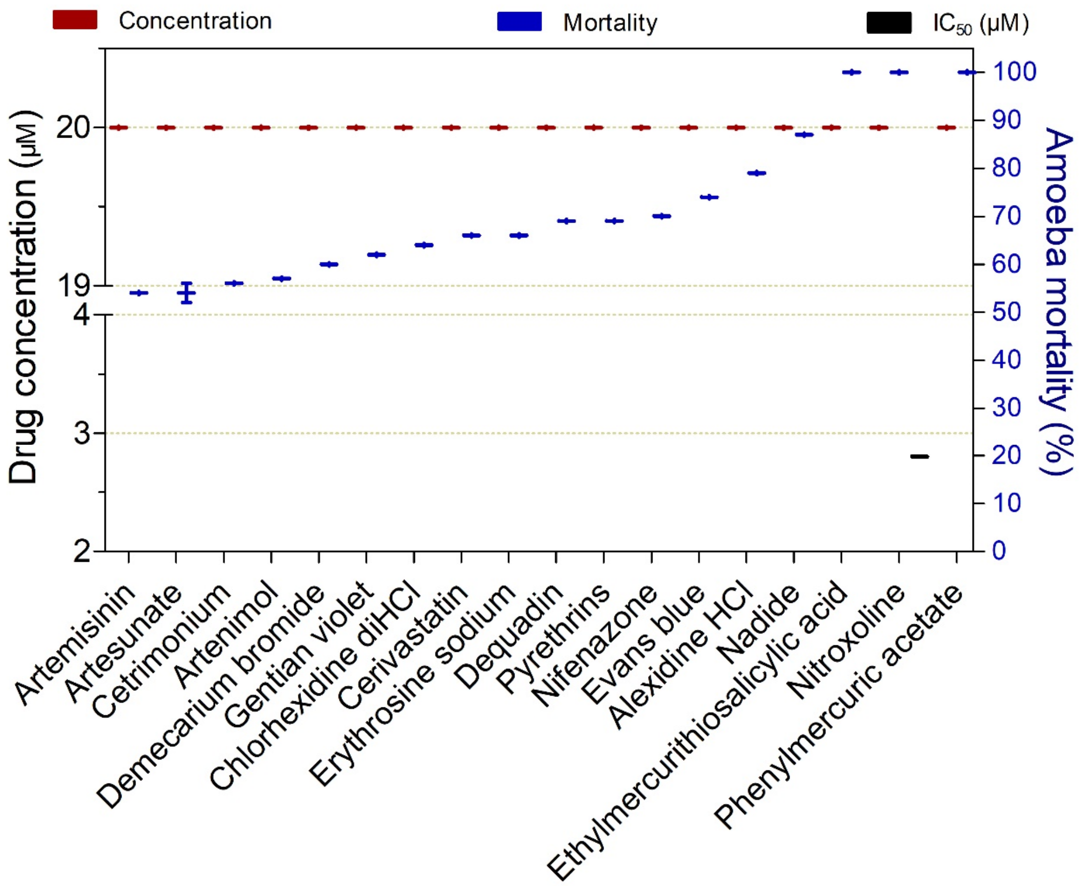


**Figure S1.** Drugs exhibiting ≥50% trophocidal activity against *Balamuthia mandrillaris* at average concentrations of 20 μM (72 h exposure time).

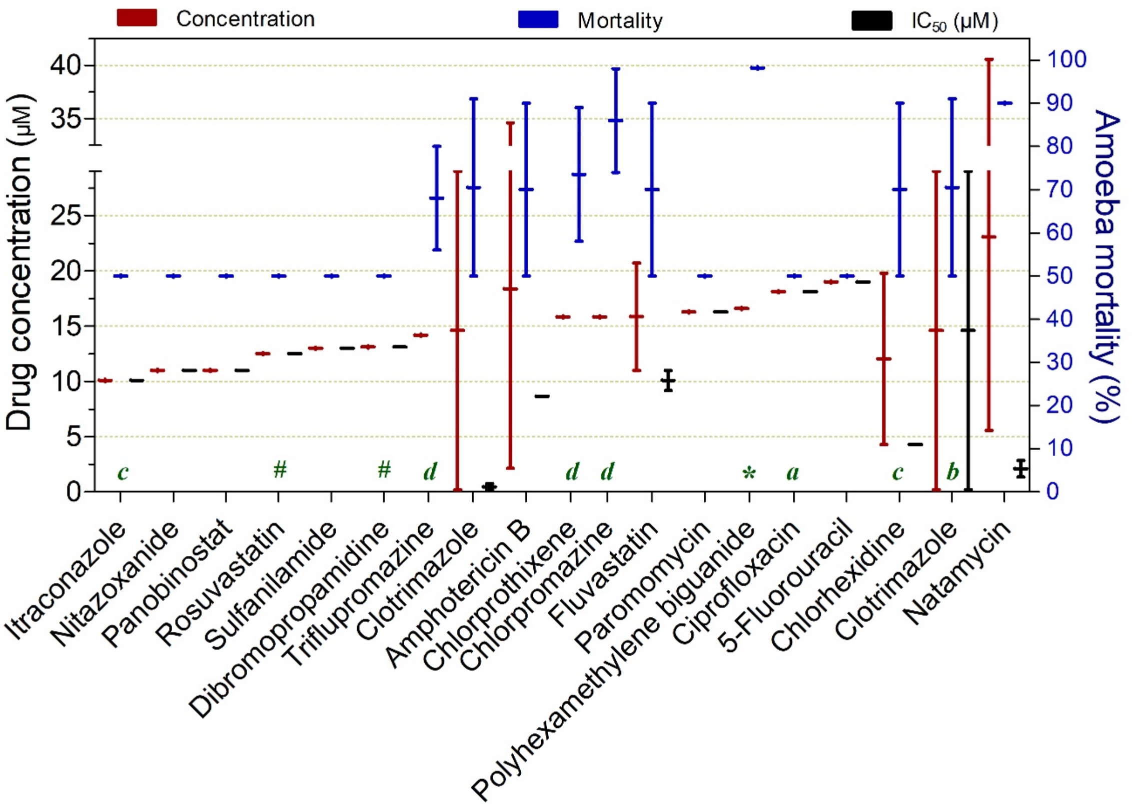


**Figure S2.** Drugs exhibiting ≥50% trophocidal activity against *Acanthamoeba* spp. at mean concentrations ranging from 10.1 to 23.06 μM. Exposure time of 72 h, except (*) – 4 h, (**) – 12 h, (#) – 48 h, (a) – 24 h, (b) – 60 h, (c) – 96 h, (d) – 120 h.

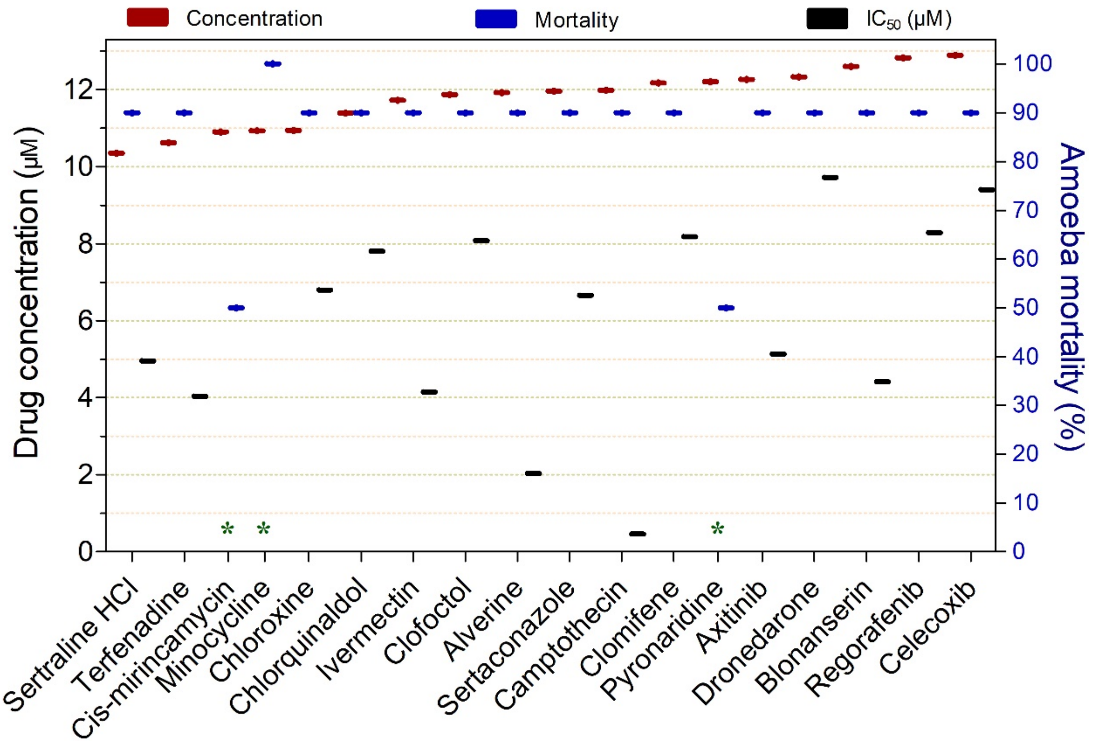

**Figure S3.** Drugs exhibiting ≥50% trophocidal activity against *Naegleria* spp. at mean concentrations ranging from 10 to 12.89 μM and an exposure time of 120 h, except (*) – 72 h.

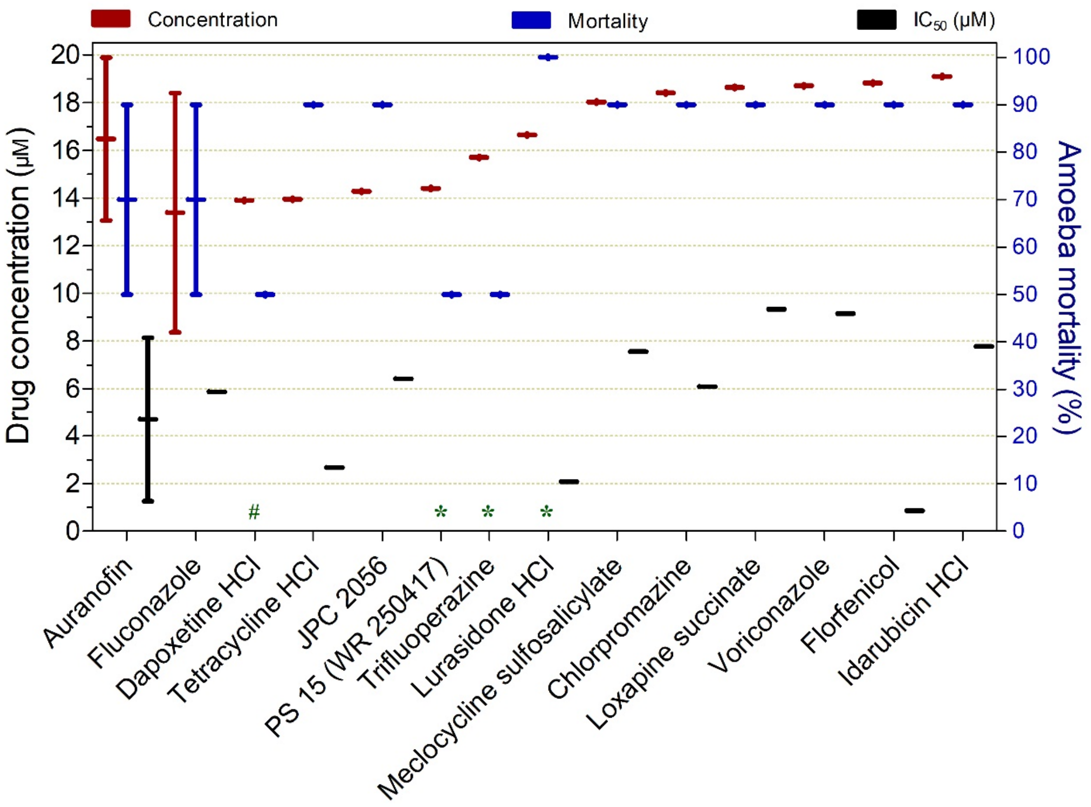


**Figure S4.** Drugs that exert a trophocidal effect ≥50% against *Naegleria* spp. at average concentrations ranging from 13 to 19,09 μM and exposure time of 120 hours, except (#) – 48 h, (*) – 72 h.

Table S1 Comparative performance of drugs tested in rat models of Granulomatous Amebic Encephalitis caused by *Acanthamoeba* spp. All compounds were administered via intraperitoneally, except those indicated by (*) administered through a subcutaneous injection

| **Drug** | **Studies (n)** | **Dose (mg/kg)** | **Treatment time (days)** | **Start of treatment dpi** | **Cure rate (%)** |
|---|---|---|---|---|---|
| Rifampicin * | 1 | 100 | 2 | 2 ** | 100 |
| Sulphadiazine | 1 | 200 | 10 | 1 | 100 |
| Rifampicin * | 1 | 100 | 5 | 2 | 88 |
| 5-Fluorocytosine * | 1 | 6 | 21 (2x/day) | 1 ** | 0 |
| Sulphamethizole | 1 | 200 | 10 | 1 | 0 |
| Sulphmethoxazole | 1 | 200 | 10 | 1 | 0 |
| Trimethoprim | 1 | 40 | 10 | 1 | 0 |

(dpi) – days post-infection. (**) – Before infection (prophylactic treatment).

Table S2. Therapeutic efficacy of drugs against *Acanthamoeba* keratitis in animal models.

| **Drug** | **n** | **Model** | **Administrative route** | **Dose (mg/kg)** | **Treatment time (days)** | **Start of treatment dpi** | **Cure rate (%)** | **Toxicity (%)** |
|---|---|---|---|---|---|---|---|---|
| Aprotinin + Neomycin (0.283mg/mL) + Atropine ointment (10) | 1 | Mouse | Eye drops | 13 | 17 (5x/day) | 2 | 100 | - |
| Chlorhexidine + neomycin sulfate + polymyxin B sulfate + gramicidin | *1* | Mouse | Eye drops | 0.02 + 4.486 + 0.5 + 0.025 | 7 (8x/day) + 21 (3x/day) | 5 | 70.5 ± 4.5 | - |
| Chlorhexidine gluconate | 1 | Mouse | Eye drops | 0.2 | 28 (8×/day/1st wk → 3×/day/2 wks) | 5 | 60.09 | 78 |
| Dexamethasone sodium phosphate | 1 | Rabbit | Intracorneal injection | 4 | 8 | 0 | 0 | - |
| Fluconazole + Neomycin (0.283) + Atropine ointment (10) | 1 | Mouse | Eye drops | 2 | 22 (5x/day) | 2 | 100 | - |
| Miltefosine | 2 | Hamsters/ Mouse | Eye drops | 0.06512 | 29 ± 1 (8×/day/1st wk → 3×/day/3 wks) | 5 | 72.5 ± 12.5 | 2 |
| Miltefosine + Propamidine isethionate | 1 | Mouse | Eye drops | 0.06512 + 0.2 | 28 (8×/day/1st wk → 3×/day/2 wks) | 5 | 70.04 | 79 |
| Miltefosine + Chlorhexidine | 1 | Mouse | Eye drops | 0.06512 + 0.2 | 28 (8×/day/1st wk → 3×/day/2 wks) | 5 | 70.04 | 77 |
| Miltefosine + Polyhexanide | 1 | Mouse | Eye drops | 0.06512 + 0.2 | 28 (8×/day/1st wk → 3×/day/2 wks) | 5 | 80.12 | 69 |
| Neomycin (0.283) + Atropine ointment (10) | 1 | Mouse | Eye drops | 13 | 28 (5x/day) | 2 | 100 | - |
| Polyhexamethylene biguanide + Neomycin (0.283) + Atropine ointment (10) | 1 | Mouse | Eye drops | 0.2 | 13 (5x/day) | 2 | 100 | - |
| Povidone iodine + Neomycin (0.283) + Atropine ointment (10) | 1 | Mouse | Eye drops | 50 | 23 (5x/day) | 2 | 100 | |
| Polyhexanide | 1 | Mouse | Eye drops | 0.2 | 28 (8×/day/1st wk → 3×/day/2 wks) | 5 | 70.04 | 65 |
| Propamidine isethionate | 1 | Mouse | Eye drops | 1.0 | 28 (8×/day/1st wk → 3×/day/2 wks) | 5 | 60.09 | 69 |
| Voriconazol | 1 | Rats | Eye drops | 10 | 21 (13x/day/3days; 7x/day/11 days and 4x/day/7days) | 7 | 88.9 | - |
| Voriconazol | 1 | Rats | Gavage | 60 mg/kg | 21 (2x/day) | 7 | 33.3 | - |
| Riboflavin + Ultraviolet A | 1 | Mouse | Eye drops | 0.1% riboflavina + UVA, 3 mW/cm$^2$ (dose of 5.4 J/cm$^2$) | 3 (5 – 30 min before UV A, → 5 min in UV A) | 3 | 0 | - |
| Polyhexamethylene biguanide | 1 | Mouse * | Eye injection | 0.2 | 1 | - | - | 100 |
| Polyhexamethylene biguanide | 1 | Mouse * | Eye injection | 0.1 | 1 | - | - | 0 |
| Propamidine isethionate | 1 | Mouse * | Eye | 1 | 1 | - | - | 100 |

| | | | | | | | | |
|---|---|---|---|---|---|---|---|---|
| | | | injection | | | | | |
| Propamidine isethionate | 1 | Mouse * | Eye injection | 0.5 | 1 | - | - | 0 |

(dpi) – days post-infection. (*) – Uninfected animals.

Table S3. Drug efficacy in mouse models of primary amoebic meningoencephalitis caused by *Naegleria fowleri*. All drugs were administered intraperitoneally.

| **Drug** | **Studies (n)** | **Dose (mg/mL)** | **Treatment time (days)** | **Start of treatment dpi** | **Cure rate (%)** | **Toxicity** |
|---|---|---|---|---|---|---|
| Amphotericin B + azithromycin | 1 | 2.5 + 25 | 5 | 3 | 100 | - |
| Cyclophosphamide | 1 | 30 | 10 | 0 | 92 | - |
| Amphotericin B + tetracycline | 1 | 2.5 + 150 | 7 | 3 | 87.5 | - |
| Rokitamycin | 1 | 20 | 3 (3x/day)* | 3 | 80 | No |
| Chlorpromazine | 1 | 20 | 3* | 3 | 75 | - |
| Azithromycin + posaconazole | 1 | 25 + 20 | 3 | 3 | 70 | - |
| Amphotericin B | 1 | 75 | 14 | 1# | 60 | - |
| Azithromycin | 1 | 25 | 5 | 3 | 55 | - |
| Miltefosine | 1 | 20 | 3* | 3 | 55 | - |
| Amphotericin B | 1 | 10 | 8 | 3 | 40 | - |
| Amphotericin B + rifamycin | 1 | 2.5 + 150 | 10 | 2 | 40 | - |
| Azithromycin | 2 | 25 | 4 ± 1 | 3 | 35 ± 5 | - |
| Posaconazole | 1 | 20 | 3 | 3 | 33 | - |
| Azithromycin + fluconazole | 1 | 25 + 30 | 3 | 3 | 30 | - |
| Azithromycin + ketoconazole | 1 | 25 + 25 | 3 | 3 | 30 | - |
| Clotrimazole | 1 | 100 | 5 | 1 | 30 | - |
| Fluconazole | 1 | 30 | 3 | 3 | 30 | - |
| Amphotericin B | 5 | 2.5 | 7 ± 3 | 3 ± 0.5 | 30 ± 15 | - |
| AN3057 | 1 | 50 | 10 (3x/day) | 1 | 28 | No |
| Roxithromycin | 1 | 20 | 3 (3x/day)* | 3 | 25 | - |
| Ketoconazole | 1 | 25 | 3 | 3 | 10 | - |
| Hygromycin B | 1 | 20 | 3 (3x/day)* | 3 | 0 | - |
| Metronidazole | 1 | 1** | 10 | 1# | 0 | - |
| Miltefosine | 1 | 20 | 3 | 3 | 0 | - |
| Pyrimethamine | 1 | 20 | 10 | 2 | 0 | Yes |
| Rifamycin | 1 | 150 | 10 | 2 | 0 | - |

(AN3057) – 4-(1-hydroxy-1,3-dihydrobenzo[c][1,2]oxaborol-5-yloxy)phenyl)methanaminium chloride. (dpi) – days post-infection. (**) – g/kg. (*) – Animals were treated on days 3, 7 and 11 after infection. (#) – Before infection (prophylactic treatment).
